# Cardiorespiratory fitness in children: Evidence for criterion-referenced cut-points

**DOI:** 10.1371/journal.pone.0201048

**Published:** 2018-08-01

**Authors:** Diego Augusto Santos Silva, Justin J. Lang, Joel D. Barnes, Grant R. Tomkinson, Mark S. Tremblay

**Affiliations:** 1 Healthy Active Living and Obesity Research Group, Children’s Hospital of Eastern Ontario Research Institute, Ottawa, Canada; 2 Research Center in Kinanthropometry and Human Performance, Federal University of Santa Catarina, Florianopolis, Brazil; 3 Public Health Agency of Canada, Ottawa, Canada; 4 Kinesiology and Public Health Education, University of North Dakota, Grand Forks, United States of America; 5 Alliance for Research in Exercise, Nutrition and Activity, University of South Australia, Adelaide, Australia; University of Oslo, NORWAY

## Abstract

**Introduction:**

Criterion-referenced cut-points for field-based cardiorespiratory fitness for children (CRF) are lacking. This study determined: (a) the association between CRF and obesity, (b) the optimal cut-points for low CRF associated with obesity in children, and (c) the association between obesity and peak oxygen uptake ($\dot{V}{\text{O}}_{2\text{peak}}$) estimated from the 20-m shuttle run test using two different prediction equations.

**Methods:**

A total of 8,740 children aged 10.1±1.2 were recruited from 11 sites across Canada. CRF was assessed using 20mSRT reported as running speed at the last completed stage, number of completed laps and predicted $\dot{V}{\text{O}}_{2\text{peak}}$, which was estimated at the age by sex level using the Léger et al. and FitnessGram equations. Body mass index and waist circumference z-scores were used to identify obesity. Receiver operating characteristic (ROC) curves and logistic regression determined the discriminatory ability of CRF for predicting obesity.

**Results:**

20mSRT had satisfactory predictive ability to detect obesity estimated by BMI, WC, and BMI and WC combined (area under the curve [AUC]>0.65). The FitnessGram equation (AUC>0.71) presented somewhat higher discriminatory power for obesity than the equation of Léger et al. (AUC>0.67) at most ages. Sensitivity was strong (>70%) for all age- and sex-specific cut-points, with optimal cut-points in 8- to 12-year-olds for obesity identified as 39 mL•kg^-1^•min^-1^ (laps: 15; speed: 9.0 km/h) and 41 mL•kg-^1^•min^-1^ (laps: 15–17; speed: 9.0 km/h) for girls and boys, respectively.

**Conclusions:**

20mSRT performance is negatively associated with obesity and CRF cut-points from ROC analyses have good discriminatory power for obesity.

## Introduction

Cardiorespiratory fitness (CRF) is an important indicator of health in children and adolescents [1]. Longitudinal cohort data indicate that low CRF in late adolescence is associated with an increased risk of all-cause mortality in adulthood [2]. CRF in childhood and adolescence has also declined since the early 1980s, suggesting a meaningful decline in population health [3].

CRF has a strong association with some cardiometabolic risk factors in children and youth [4–6]. On the other hand, there is an association between fatness and cardiometabolic risk factors in youth, such that as fatness increases the risk of an unfavorable metabolic risk profile also increases [4]. The extent to which adjustment for adiposity attenuates or modifies the association between CRF and metabolic risk is uncertain. In this sense, to examine the magnitude of association between obesity and CRF at the population level may help to better understand the causal pathway of the relationship between CRF, obesity, and cardiometabolic risk factors [4].

CRF can be measured using laboratory-based tests (e.g., indirect calorimetry using expired gas analyses) or field-based tests. The use of laboratory-based tests in school- and population-based studies is limited due to their high cost, necessity of sophisticated instruments, technical expertise requirements and time constraints [7]. Field-based tests provide a practical alternative since they have logistical advantages including increased feasibility, low cost, and ease of administration to a large number of people simultaneously while maintaining acceptable accuracy [1,8].

One of the most common field tests for assessing CRF in young people is the 20-m shuttle run test (20mSRT) [9,10]. This test was originally described in 1984 by Léger et al. [6,7] and is the most widely used field test of CRF with normative data available for over 1.1 million children and youth from 50 countries [1,8,11,12]. Two recent reviews with data from children and youth reported that the 20mSRT performance could accurately identify health risk factors [6,13] demonstrating its utility as an assessment of population health for children and adolescents.

A challenge of studies that relate CRF with health indicators is to define the cut-points for CRF tests capable of adequately discriminating between healthy and unhealthy individuals [7]. A recent review identified that only 10 studies published between 2006 and 2016 defined criterion-referenced cut-points for CRF tests in children and adolescents [13]. While half of these studies used the 20mSRT to evaluate CRF, most used local or state/provincial samples that may not have been population representative [8]. Furthermore, while criterion-referenced cut-points for CRF exist for many high-income countries, none have yet been identified among Canadian children. In addition, it is necessary to verify whether cut-points from different populations converge in order to inform potential international criterion-referenced standards.

Another challenge for those working with the 20mSRT is to select an equation to predict $\dot{V}{\text{O}}_{2\text{peak}}$. The original authors of the test proposed an equation with validity for the paediatric population (9–19 years) [9,10]. However, different equations for the same age group have been proposed after the creation of the test by authors from different regions of the world in order to better estimate the $\dot{V}{\text{O}}_{2\text{peak}}$ of children and adolescents [14–18]. FitnessGram^®^ (http://www.cooperinstitute.org/fitnessgram) experts discussed this lack of consensus of equations to estimate $\dot{V}{\text{O}}_{2\text{peak}}$ using 20mSRT [19] and concluded that the use of indicators such as body mass, height, BMI or fat percentage as constants in predictive equations, although improving the prediction of $\dot{V}{\text{O}}_{2\text{peak}}$, are not recommended by FitnessGram^®^ because CRF and body composition are distinct physical fitness components [19]. Although they are related to each other, the inclusion of one as a predictive variable for the other demonstrates codependence, violating the belief that all components of fitness are assumed to be equally important [19]. Furthermore, the use of measures additional to 20mSRT increases the burden of the test, as it requires greater logistics for data collection, such as body mass and height assessment. As a result, FitnessGram^®^ makes use of an equation to estimate $\dot{V}{\text{O}}_{2\text{peak}}$ that no longer takes into account measures of body composition [15]. Thus, comparing the differences in the criterion-referenced standards for different predictive equations used to estimate $\dot{V}{\text{O}}_{2\text{peak}}$ will help identify the most appropriate equation and obesity cut-point for CRF among Canadian children.

The aim of this study was to determine the association between CRF estimated by the 20mSRT and obesity to determine population representative criterion-referenced cut-points in a large, diverse sample of Canadian children. In addition, this study compared the associations between obesity and $\dot{V}{\text{O}}_{2\text{peak}}$ using estimates from the Léger et al. [9] and FitnessGram^®^ [15] equations.

## Materials and methods

### Study design

This study is part of the RBC Learn to Play—Canadian Assessment of Physical Literacy (RBC-CAPL) project [20]. RBC-CAPL is a cross-sectional surveillance study that was carried out between 2014 and 2016, designed to evaluate the physical literacy levels of Canadian children using a standardized data collection protocol. The study included 11 data collection sites from seven Canadian provinces including Victoria (British Columbia), Lethbridge (Alberta), Calgary (Alberta), Winnipeg (Manitoba), North Bay (Ontario), Windsor (Ontario), Ottawa (Ontario), Trois-Rivières (Québec), Halifax (Nova Scotia), Antigonish (Nova Scotia), and Charlottetown (Prince Edward Island). The aim was to recruit up to 1,300 participants per site over a 3-year data collection period. Each site was also tasked with recruiting participants from both urban (minimum of 50% of the sample) and rural (minimum of 20% of the sample) locations, while ensuring a balanced representation of high-, medium- and low-income communities. Ethics approval was obtained from: Antigonish—St. Francis University Research Ethics Board and the Strait Regional School Board; Calgary—Mount Royal University Human Research Ethics Board; Charlottetown—University of Prince Edward Island Research Ethics Board and the Prince Edward Island Public Schools Branch Research Ethics Board; Halifax—Dalhousie University Research and Ethics Board and the Halifax Regional School Board; Lethbridge—University of Lethbridge Human Subject Research Committee; North Bay—Nipping University Research Ethics Board, Near North District School Board, Nipissing Parry Sound Catholic District School Board, and Conseil Scolaire Catholique Franco-Nord; Ottawa—Children’s Hospital of Eastern Ontario Research Ethics Board, University of Ottawa Research Ethics Board, Ottawa-Carleton District School Board, Ottawa Catholic School Board, Conseil des écoles catholiques du Centre-Est, Conseil des écoles publiques de l’Est de l’Ontario, Upper Canada District School Board, Durham District School Board, University of Illinois at Urbana-Champaign; Trois-Rivières—Université du Québec à Trois-Rivières Research Ethics Board; Victoria—Camosun College Research Ethics Board and the Greater Victoria School District; Windsor—University of Windsor Research Ethics Board and the Windsor Essex Catholic District School Board; Winnipeg—The University of Winnipeg University Human Research Ethics Board (UHREB), River East Transcona School Division, and St. James-Assiniboia School Division. Written informed consent was obtained from parents or legal guardians, and child assent was also obtained.

### Participants

Participant recruitment locations were selected across all sites using purposive, non-randomized sampling. Elementary schools across all sites were the primary participant recruitment locations for this study, whereas summer camps, community centres and sport leagues were the secondary participant recruitment locations. Participants were considered eligible for this study if they were aged 8.0–12.9 years (grades 4–6), and maximal effort exercise was not contraindicated. All eligible participants were invited to participate in this study, but potential participants were able to drop out for any reason, without consequence. Of the 10,034 participants who took part in RBC-CAPL, a total of 8,740 remained in the present analysis after excluding participants without a 20mSRT score (n = 641), body mass index (BMI; n = 323) and waist circumference (WC; n = 184) values. This study used control variables in the analyses, so subjects without information on screen time (n = 137) and physical activity (n = 9) were excluded.

### Data collection procedures

Data collection staffs had a background in fitness or physical activity assessment, and were subsequently trained by research staff from the coordinating centre (Ottawa). Data collection procedures followed the published CAPL protocol [21,22], which provided standardized procedures to collect data across the four physical literacy domains: physical competence, daily behaviour, knowledge and understanding, and motivation and confidence.

### Cardiorespiratory fitness measures

CRF was assessed using the 15 m or 20mSRT protocols [9,10]. All children were asked to run back and forth between two parallel lines, 15 m or 20 m apart, following the pace of an audio signal that began at a speed of 8.5 km/h and increased by 0.5 km/h at 1-minute intervals. The number of laps (shuttles) completed was recorded for each participant, and all data from the 15-m protocol were converted into the 20-m protocol using a conversion chart [23]. Researchers used indoor gymnasiums as the primary testing location, with outdoor locations used as a back-up. Participants were encouraged at all times to produce a maximal effort. Following the Tomkinson et al. [11] recommendations, 20mSRT performance was reported as $\dot{V}{\text{O}}_{2\text{peak}}$, the running speed at the last completed stage, and the number of completed laps.

$\dot{V}{\text{O}}_{2\text{peak}}$ was estimated using both the Léger et al. [9,10] and FitnessGram^®^ [15] equations. Both equations adopted in this study are easily applied and do not require anthropometric information or body composition as is the case with other equations [14,17,18]. This allows the practical application of the test, considering that all these equations present low standard errors of estimate when predicting $\dot{V}{\text{O}}_{2\text{peak}}$ [9,10,15]. Moreover, the use of both equations in the present study minimizes the impact of BMI and physical growth indicators on $\dot{V}{\text{O}}_{2\text{peak}}$ estimates [19,24,25]. To compare the cut-off points established in this study with those from other studies, the corresponding metabolic equivalent (METs) was also calculated by dividing $\dot{V}{\text{O}}_{2\text{peak}}$ by 3.5 mL•kg^-1^•min^-1^ [7].

### Obesity measures

Obesity was estimated using BMI and WC information. Height was measured to the nearest 0.1 cm using a stadiometer. Body mass was recorded to the nearest 0.1 kg using a digital weighing scale. Both measures were reported as the average of two measurements, or if duplicate measurements differed by more than 0.5 cm or 0.5 kg, the average of the closest two of three measures. BMI (kg/m^2^) was subsequently derived and BMI z-scores calculated using age- and sex-specific reference data from the World Health Organization, with obesity defined as >+2 standard deviation [26].

WC was measured at the superior iliac crest at the end of a normal expiration, and reported as the average of two measures, or if duplicate measurements differed by more than 0.5 cm, the average of the closest two of three measures [22]. Age- and sex-specific WC z-scores were calculated, with obesity defined as >+2 standard deviation. This strategy was chosen because there are no specific cut-points to define abdominal obesity in this age group.

After defining obesity using BMI z-score and WC z-score, a new variable was generated from the combination of both. Children were classified as obese simultaneously by the BMI z-score and WC z-score. This strategy was used because both measures (BMI and WC) are less accurate indicators than imaging or densitometric techniques for the diagnosis of obesity and therefore a subject may be obese using BMI z-score and non-obese using WC z-score, or vice versa [27]. In this sense, the combination of both measures increases the chances of identifying participants with excess body fat [27].

### Control variables

Control variables included self-reported age (whole years), city of residence, screen time (i.e., time spent using screens [e.g., watching television, playing video games, computer games, or other screen-based devices] on a typical school and weekend day) and level of physical activity (average number of days per week that they achieved at least 60 minutes of moderate to vigorous-intensity physical activity [MVPA]).

### Statistical analysis

Descriptive statistics of CRF and all variables are presented as means and standard deviations, or percentages, where appropriate. The effect size of the comparisons between the sexes was calculated (Cohen’s D for continuous variables or Cramer’s V for categorical variables). Pearson correlations were calculated to quantify the relationship between CRF and obesity. Receiver-operating characteristics (ROC) curves were calculated to examine the discriminatory ability of CRF to predict obesity quantified by the area under the curve (AUC) [28]. ROC curves were plotted using sensitivity and specificity measures based on CRF cut-points. ROC curves demonstrate the overall discriminatory power of a diagnostic test over the whole range of testing values. A better test shows its curve skewed closer to the upper left corner [29]. The area under the curve (AUC) is a measure of the diagnostic power of a test. A diagnostic test with AUC value equal to 1 is perfectly accurate, whereas a value equal to 0.5 has no discrimination power. The literature does not provide consensus on what would be the best classifications for AUCs [30,31]. However, AUCs values of 0.55–0.62, 0.63–0.71 and >0.71 corresponded to an effect size (Cohen’s d) small, medium and large, respectively [31]. Statistical significance of differences in AUCs between predictive equations to estimate $\dot{V}{\text{O}}_{2\text{peak}}$ was assessed by using the nonparametric approach of DeLong et al. [32]. Sensitivity, specificity, positive predictive value (PPV), negative predictive value (NPV), positive likelihood ratio (LR+), and negative likelihood ratio (LR-) of the CRF were calculated at all possible cut-points to find the optimal value. Optimal sensitivity and specificity were the values yielding maximum AUC from the ROC curves. The optimal value was considered the cut-point with the fewest false positives and negatives [33]. The classification error of the ROC curve was non-differential and therefore did not have co-variables.

In addition, the present sample was classified according to the cut-points suggested in the present study for CRF. This classification took into account age-specific and sex-specific cut-points and those specific only for sex. This classification allowed assessment of the association between low levels of CRF and obesity through logistic regression analysis with odds ratio (OR) and 95% confidence intervals (95% CI). For this, univariate and multivariate analyses were performed. Multivariate analyses were adjusted for age, city of residence, screen time, and level of physical activity. All analyses were performed separately for boys and girls. Statistical programs MedCalc 16.8.4^®^ (Ostend, Belgium) and Stata 13.0^®^ (College Station, USA) were used for all analyses.

## Results

The final sample comprised 8,740 children aged 10.1±1.2 years who spent an average of 2.4±1.9 hours/day on sedentary behavior-based screen time and 298±115 min/week on MVPA. Means±SDs for the total sample were: BMI, 19±4 kg/m^2^; WC, 67±11 cm; 20mSRT, 23±14 laps, 45±4 mL•kg^-1^•min^-1^ and 12.8±1.2 METs using the Léger et al. [9] equation, or 43±5 mL•kg^-1^•min^-1^ and 12.2±1.4 METs using the FitnessGram^®^ equation [15]. The prevalence of obesity according to BMI was 15.1%, the prevalence of abdominal obesity according to WC was 5.0%, and the simultaneous prevalence of obesity by BMI and WC was 4.5% (Table 1). Across all age and sex groups, CRF was a weak to moderate negative correlate of BMI and WC (S1 Table).

**Table 1:** Characteristics of the sample.

	Full sample (n = 8,740)	Boys (n = 4,369)	Girls (n = 4,371)	p	Effect size
	Mean (SD)	Mean (SD)	Mean (SD)		
**Age**	10.1 (1.2)	10.1 (1.2)	10.1 (1.2)	0.42	0.00†
**Weight (kg)**	39.9 (11.4)	39.7 (11.6)	40.0 (11.3)	0.17	0.02†
**Height (cm)**	144.00 (9.8)	143.8 (9.6)	144.2 (10.1)	0.07	0.04†
**Screen time (h/day)**	2.4 (1.9)	2.7 (2.1)	2.2 (1.7)	<0.01*	0.26†
**MVPA (min/week)**	298.0 (114.9)	302.5 (118.4)	293.5 (111.2)	<0.01*	0.07†
**BMI (kg/m**^**2**^**)**	18.9 (3.8)	18.9 (3.8)	19.0 (3.7)	0.44	0.02†
**Waist circumference (cm)**	67.3 (10.7)	67.4 (10.9)	67.2 (10.5)	0.41	0.01†
**Laps (n)**	23 (14)	26 (16)	21 (11)	<0.01*	0.36†
**$\dot{V}O_{2peak}$ (mL•kg**^**-1**^**•min**^**-1**^**)–Léger et al.**	44.7 (4.2)	45.4 (4.5)	44.0 (3.6)	<0.01*	0.34†
**$\dot{V}O_{2peak}$ (mL•kg**^**-1**^**•min**^**-1**^**)–FitnessGram**	42.6 (5.0)	43.5 (5.6)	41.6 (4.1)	<0.01*	0.38†
**METs–Léger et al.**	12.8 (1.2)	12.9 (1.3)	12.5 (1.0)	<0.01*	0.34†
**METs–FitnessGram**	12.2 (1.4)	12.4 (1.6)	11.9 (1.2)	<0.01*	0.35†
	**n (%)**	**n (%)**	**n (%)**		
**Age (years)**					
8	980 (11.2)	493 (11.3)	487 (11.1)	0.81	0.01‡
9	1,718 (19.7)	872 (20.0)	846 (19.4)		
10	2,221 (25.4)	1,119 (25.6)	1,102 (25.2)		
11	2,852 (32.6)	1,399 (32.0)	1,453 (33.2)		
12	969 (11.1)	486 (11.1)	483 (11.1)		
**BMI z-score (WHO)**					
No obesity (<+2SD)	7,423 (84.9)	3,598 (82.4)	3,825 (87.5)	<0.01*	0.07‡
Obesity (≥+2SD)	1,317 (15.1)	771 (17.6)	546 (12.5)		
**Waist circumference**					
No obesity (<+2SD)	8,302 (95.0)	4,141 (94.8)	4,161 (95.2)	0.37	0.01‡
Obesity (≥+2SD)	438 (5.0)	228 (5.2)	210 (4.8)		
**BMI and waist circumference**					
No obesity	8,346 (95.5)	4,160 (95.2)	4,186 (95.8)	0.21	0.01‡
Obesity	394 (4.5)	209 (4.8)	185 (4.2)		

SD.: standard deviation; BMI: body mass index; MVPA: moderate-to-vigorous physical activity; WHO: World Health Organization;

*p<0.01;

^†^Cohen’s *d*;

^‡^Cramer’s V.

**Fig 1:**
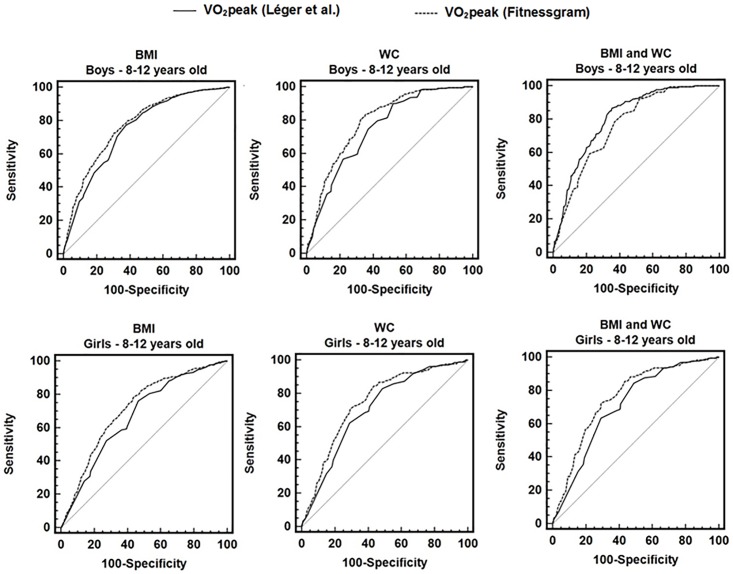
Receiver operating characteristic curve of $\dot{V}{\text{O}}_{2\text{peak}}$ estimated by the Léger et al. and FitnessGram equations to detect obesity in boys and girls according to body mass index (BMI), waist circumference (WC), and the combination between BMI and WC.

For boys (Table 2, Fig 1) and girls (Table 3, Fig 1), $\dot{V}{\text{O}}_{2\text{peak}}$ showed significant predictive capacity for obesity (AUCs>0.65), with AUCs of the FitnessGram^®^ somewhat higher than those of Léger. The best $\dot{V}{\text{O}}_{2\text{peak}}$ cut-points to detect obesity estimated by BMI, WC or by combination of BMI and WC were higher using the Léger et al equation [9] when compared to the FitnessGram^®^ equation [15] at most ages.

**Table 2:** Diagnostic properties of $\dot{V}{\text{O}}_{2\text{peak}}$ (20-meter shuttle run test) to detect obesity in boys, according to equations of Léger et al. and FitnessGram.

Boys	AUC (95% CI)	Cut-points (mL•kg^-1^•min^-1^)	Cut-points (METs)	Sensitivity (%) (95% CI)	Specificity (%) (95% CI)	PPV (%)	NPV (%)	LR+	LR-
	**Obesity by BMI z-score**								
**8 years old**									
Léger et al.	0.71 (0.67–0.75)	45.2	12.9	68.9 (55.7–80.1)	64.2 (59.6–68.7)	20.8	93.8	1.9	0.5
FitnessGram	0.74 (0.70–0.78)*	42.3	12.1	73.8 (60.9–84.2)	61.7 (57.1–66.3)	20.8	93.8	1.9	0.4
**9 years old**									
Léger et al.	0.77 (0.74–0.80)	43.4	12.5	72.2 (64.8–78.8)	73.4 (70.0–76.6)	38.6	91.9	2.7	0.4
FitnessGram	0.80 (0.76–0.82)**	40.8	11.7	72.2 (64.8–78.8)	73.4 (70.0–76.6)	38.6	91.9	2.7	0.4
**10 years old**									
Léger et al.	0.73 (0.71–0.76)	41.5	11.9	60.2 (53.2–66.8)	72.6 (69.6–75.4)	33.3	88.9	2.2	0.6
FitnessGram	0.76 (0.73–0.79)**	41.1	11.8	76.8 (70.5–82.3)	60.5 (57.2–63.6)	30.7	92.0	1.9	0.4
**11 years old**									
Léger et al.	0.77 (0.75–0.79)	39.7	11.3	66.1 (60.1–71.7)	77.8 (75.2–80.1)	41.3	90.6	2.9	0.4
FitnessGram	0.80 (0.77–0.82)**	39.6	11.3	77.1 (71.7–82.0)	68.2 (65.4–70.9)	36.5	92.6	2.4	0.3
**12 years old**									
Léger et al.	0.80 (0.76–0.83)	40.3	11.5	81.6 (71.0–89.5)	63.6 (58.8–68.2)	28.7	95.1	2.2	0.3
FitnessGram	0.80 (0.76–0.84)	40.3	11.5	81.6 (71.0–89.5)	63.6 (58.8–68.2)	28.7	95.1	2.2	0.3
**8–12 years old**									
Léger et al.	0.75 (0.74–0.76)	43.9	12.5	77.4 (74.3–80.3)	62.3 (60.7–63.9)	30.6	92.8	2.1	0.4
FitnessGram	0.77 (0.76–0.79)**	40.5	11.6	72.3 (69.1–75.4)	69.7 (68.2–71.2)	33.9	92.1	2.4	0.4
	**Obesity by waist circumference z-score**								
**8 years old**									
Léger et al.	0.74 (0.70–0.77)	45.2	12.9	71.4 (53.7–85.3)	62.6 (58.0–67.0)	12.4	96.7	1.9	0.5
FitnessGram	0.77 (0.73–0.81)*	42.3	12.1	77.1 (59.9–89.5)	62.6 (58.0–67.0)	12.5	97.3	1.9	0.4
**9 years old**									
Léger et al.	0.83 (0.80–0.85)	43.4	12.4	90.7 (77.8–97.3)	67.6 (64.4–70.7)	12.3	99.3	2.8	0.1
FitnessGram	0.86 (0.84–0.88)**	40.1	11.5	90.7 (77.8–97.3)	75.7 (72.7–78.5)	15.8	99.4	3.7	0.1
**10 years old**									
Léger et al.	0.77 (0.75–0.79)	41.5	11.9	73.5 (61.4–83.5)	69.0 (66.2–71.8)	13.1	97.6	2.4	0.4
FitnessGram	0.81 (0.79–0.83)**	40.4	11.6	85.3 (74.6–92.7)	62.7 (59.7–65.6)	12.7	98.5	2.3	0.2
**11 years old**									
Léger et al.	0.80 (0.77–0.82)	39.7	11.3	83.1 (71.7–91.2)	71.9 (69.4–74.3)	12.5	98.9	2.9	0.2
FitnessGram	0.81 (0.79–0.83)	38.6	11.0	83.1 (71.7–91.2)	71.9 (69.4–74.3)	12.5	98.9	2.9	0.2
**12 years old**									
Léger et al.	0.82 (0.78–0.85)	37.8	10.8	73.9 (51.6–89.7)	78.2 (74.2–81.8)	14.0	98.4	3.4	0.3
FitnessGram	0.84 (0.80–0.87)*	38.2	10.9	82.6 (61.2–94.9)	74.2 (70.0–78.0)	13.4	98.9	3.2	0.2
**8–12 years old**									
Léger et al.	0.75 (0.74–0.76)	43.4	12.4	74.8 (68.7–80.2)	62.9 (61.4–64.3)	10.0	97.8	2.0	0.4
FitnessGram	0.79 (0.78–0.81)**	40.6	11.6	83.8 (78.4–88.2)	64.5 (63.0–66.0)	11.6	98.6	2.4	0.3
	**Obesity by BMI and waist circumference**								
**8 years old**									
Léger et al.	0.75 (0.71–0.79)	45.2	12.9	76.9 (56.3–91.0)	62.2 (57.7–66.6)	9.9	98.0	2.0	0.4
FitnessGram	0.78 (0.74–0.82)	42.0	12.0	76.9 (56.3–91.0)	62.2 (57.7–66.6)	9.9	98.0	2.0	0.4
**9 years old**									
Léger et al.	0.83 (0.80–0.85)	43.4	12.4	90.7 (77.8–97.3)	67.6 (64.4–70.7)	12.3	99.3	2.8	0.1
FitnessGram	0.86 (0.84–0.88)**	40.1	11.5	90.7 (77.8–97.3)	75.7 (72.7–78.5)	15.8	99.4	3.7	0.1
**10 years old**									
Léger et al.	0.78 (0.76–0.81)	41.5	11.9	75.0 (62.6–85.0)	69.0 (66.1–71.7)	12.6	97.9	2.4	0.4
FitnessGram	0.82 (0.80–0.84)**	40.4	11.6	87.5 (76.8–94.4)	62.6 (59.7–65.5)	12.3	98.8	2.3	0.2
**11 years old**									
Léger et al.	0.82 (0.80–0.84)	39.7	11.3	86.9 (75.8–94.1)	71.9 (69.4–74.3)	12.2	99.2	3.1	0.2
FitnessGram	0.84 (0.82–0.86)	38.6	11.0	86.9 (75.8–94.1)	71.9 (69.4–74.3)	12.2	99.2	3.1	0.2
**12 years old**									
Léger et al.	0.82 (0.78–0.85)	37.8	10.8	76.2 (52.8–91.7)	78.0 (74.0–81.7)	13.2	98.7	3.5	0.3
FitnessGram	0.84 (0.81–0.87)*	38.2	10.9	81.0 (58.1–94.4)	73.8 (69.7–77.7)	12.0	98.9	3.1	0.3
**8–12 years old**									
Léger et al.	0.77 (0.75–0.78)	43.4	12.4	78.6 (72.5–83.9)	62.9 (61.4–64.3)	9.7	98.3	2.1	0.3
FitnessGram	0.81 (0.80–0.82)**	40.6	11.6	87.0 (81.7–91.2)	63.9 (62.4–65.4)	10.9	99.0	2.4	0.2

BMI: body mass index; AUC: area under the curve; 95% CI: 95% confidence interval; PPV: positive predictive value; NPV: negative predictive value; LR+: positive likelihood ratio; LR: negative likelihood ratio;

*Significant difference (p < 0.05) compared with the information of the Leger et al. equation;

**Significant difference (p < 0.05) compared with the information of the Leger et al. equation.

**Table 3:** Diagnostic properties of $\dot{V}{\text{O}}_{2\text{peak}}$ (20-meter shuttle run test) to detect obesity in girls, according to equations of Léger et al. and FitnessGram.

Girls	AUC (95% CI)	Cut-points (mL•kg^-1^•min^-1^)	Cut-points (METs)	Sensitivity (%) (95% CI)	Specificity (%) (95% CI)	PPV (%)	NPV (%)	LR+	LR-
	**Obesity by BMI z-score**								
**8 years old**									
Léger et al.	0.66 (0.61–0.70)	45.2	12.9	73.6 (59.7–84.7)	52.5 (47.7–57.2)	15.5	94.4	1.6	0.5
FitnessGram	0.69 (0.64–0.73)	40.9	11.7	62.3 (47.9–75.2)	65.6 (61.0–70.0)	17.6	93.6	1.8	0.6
**9 years old**									
Léger et al.	0.72 (0.69–0.75)	43.4	12.4	78.9 (71.0–85.5)	60.8 (57.1–64.3)	26.8	94.1	2.0	0.4
FitnessGram	0.75 (0.72–0.78)*	40.8	11.7	78.9 (71.0–85.5)	60.8 (57.1–64.3)	26.8	94.1	2.0	0.4
**10 years old**									
Léger et al.	0.68 (0.65–0.71)	41.5	11.9	74.0 (66.2–80.8)	59.5 (56.3–62.6)	22.2	93.6	1.8	0.4
FitnessGram	0.72 (0.69–0.75)**	40.1	11.4	80.0 (72.7–86.1)	54.9 (51.7–58.1)	21.7	94.6	1.8	0.4
**11 years old**									
Léger et al.	0.72 (0.70–0.75)	39.7	11.3	69.6 (62.1–76.5)	68.6 (66.0–71.2)	22.4	94.6	2.2	0.4
FitnessGram	0.74 (0.72–0.76)	39.3	11.2	78.6 (71.6–84.5)	61.1 (58.4–63.8)	20.8	95.6	2.0	0.4
**12 years old**									
Léger et al.	0.73 (0.69–0.77)	37.8	10.8	61.2 (46.2–74.8)	73.9 (69.6–77.9)	20.4	94.6	2.4	0.5
FitnessGram	0.77 (0.73–0.80)**	38.2	10.9	75.5 (61.1–86.6)	67.0 (62.5–71.4)	20.0	96.2	2.3	0.4
**8–12 years old**									
Léger et al.	0.67 (0.66–0.69)	43.0	12.3	60.6 (56.4–64.7)	60.3 (58.7–61.8)	17.9	91.5	1.5	0.7
FitnessGram	0.72 (0.70–0.73)**	40.1	11.4	72.9 (69.0–76.5)	60.5 (58.9–62.0)	20.8	94.0	1.8	0.5
	**Obesity by waist circumference z-score**								
**8 years old**									
Léger et al.	0.78 (0.74–0.82)	45.2	12.9	88.9 (65.2–98.3)	51.1 (46.6–55.7)	6.3	99.2	1.8	0.2
FitnessGram	0.79 (0.75–0.82)	40.5	11.6	77.8 (52.4–93.5)	73.5 (69.3–77.4)	9.9	98.9	2.9	0.3
**9 years old**									
Léger et al.	0.76 (0.73–0.79)	43.4	12.4	86.5 (71.2–95.4)	56.5 (53.1–59.9)	8.2	98.9	2.0	0.2
FitnessGram	0.80 (0.77–0.83)**	40.5	11.6	81.1 (64.8–92.0)	63.3 (59.9–66.6)	9.0	98.7	2.2	0.3
**10 years old**									
Léger et al.	0.70 (0.67–0.72)	41.5	11.9	81.0 (69.1–89.7)	57.1 (54.1–60.2)	10.2	98.0	1.9	0.3
FitnessGram	0.76 (0.74–0.79)**	39.0	11.1	73.0 (60.3–83.4)	70.9 (68.1–73.6)	13.1	97.8	2.5	0.4
**11 years old**									
Léger et al.	0.72 (0.70–0.75)	39.7	11.3	73.2 (61.4–83.1)	66.1 (63.6–68.6)	10.0	98.0	2.2	0.4
FitnessGram	0.74 (0.72–0.76)	38.9	11.1	80.3 (69.1–88.8)	62.3 (59.7–64.9)	9.8	98.4	2.1	0.3
**12 years old**									
Léger et al.	0.69 (0.64–0.73)	40.3	11.5	87.5 (67.6–97.2)	43.5 (38.9–48.1)	7.3	98.6	1.6	0.3
FitnessGram	0.71 (0.67–0.75)	38.2	10.9	70.8 (48.9–87.3)	64.6 (60.1–68.9)	9.2	97.8	2.0	0.5
**8–12 years old**									
Léger et al.	0.71 (0.69–0.72)	42.1	12.0	68.5 (61.8–74.7)	62.1 (60.7–63.6)	8.4	97.5	1.8	0.5
FitnessGram	0.75 (0.73–0.76)**	39.2	11.2	70.9 (64.3–76.9)	70.7 (69.3–72.1)	10.9	98.0	2.4	0.4
	**Obesity by BMI and waist circumference**								
**8 years old**									
Léger et al.	0.78 (0.74–0.82)	45.2	12.9	88.2 (63.5–98.2)	51.0 (46.5–55.6)	6.0	99.2	1.8	0.2
FitnessGram	0.78 (0.74–0.82)	40.5	11.6	76.5 (50.1–93.0)	73.3 (69.2–77.2)	9.2	98.9	2.9	0.3
**9 years old**									
Léger et al.	0.77 (0.74–0.80)	43.4	12.4	90.9 (75.6–98.0)	56.5 (53.0–59.9)	7.7	99.4	2.1	0.2
FitnessGram	0.81 (0.78–0.83)*	40.5	11.6	84.8 (68.1–94.8)	63.2 (59.8–66.5)	8.4	99.1	2.3	0.2
**10 years old**									
Léger et al.	0.69 (0.66–0.71)	41.5	11.9	80.0 (67.7–89.2)	57.0 (53.9–60.0)	9.6	98.0	1.9	0.4
FitnessGram	0.76 (0.73–0.78)**	39.0	11.1	73.3 (60.3–83.9)	70.8 (67.9–73.5)	12.6	97.9	2.5	0.4
**11 years old**									
Léger et al.	0.76 (0.74–0.78)	39.7	11.3	80.7 (68.1–89.9)	66.0 (63.5–68.5)	8.8	98.8	2.4	0.3
FitnessGram	0.79 (0.76–0.81)	38.6	11.0	80.7 (68.1–89.9)	66.0 (63.5–68.5)	8.8	98.8	2.4	0.3
**12 years old**									
Léger et al.	0.69 (0.65–0.73)	40.3	11.5	90.0 (68.3–98.5)	43.3 (38.8–47.9)	6.2	99.0	1.6	0.2
FitnessGram	0.73 (0.69–0.77)*	38.2	10.9	75.0 (50.9–91.2)	64.4 (60.0–68.7)	8.1	98.4	2.1	0.4
**8–12 years old**									
Léger et al.	0.71 (0.70–0.72)	42.1	12.0	67.9 (60.7–74.5)	61.9 (60.4–63.4)	7.3	97.8	1.8	0.5
FitnessGram	0.76 (0.75–0.77)**	39.2	11.2	72.7 (65.7–79.0)	70.5 (69.1–71.9)	9.8	98.3	2.5	0.4

BMI: body mass index; AUC: area under the curve; 95% CI: 95% confidence interval; PPV: positive predictive value; NPV: negative predictive value; LR+: positive likelihood ratio; LR: negative likelihood ratio;

*Significant difference (p < 0.05) compared with the information of the Leger et al. equation;

**Significant difference (p < 0.05) compared with the information of the Leger et al. equation.

In boys, when considering the full sample (8–12 years), the optimal cut-point for $\dot{V}{\text{O}}_{2\text{peak}}$ estimated using the Léger et al. equation [9] to detect obesity by combining BMI and WC was 43 mL•kg^-1^•min^-1^ (sensitivity of 79% and specificity of 63%). For $\dot{V}{\text{O}}_{2\text{peak}}$ estimated using the FitnessGram^®^ equation [15], the optimal cut-point for detecting obesity by combining BMI and WC in the full sample (8–12 years) was 41 mL•kg^-1^•min^-1^ (sensitivity of 87% and specificity of 64%). Table 2, Fig 1 and S1, S2 and S3 Figs show information on AUCs, the optimal cut-points of $\dot{V}{\text{O}}_{2\text{peak}}$ for detecting obesity, and diagnostic measures for each age.

For girls, when considering the full sample (8–12 years), the optimal cut-point estimated using the Léger et al. equation [9] to detect obesity by combining BMI and WC was 42 mL•kg^-1^•min^-1^ (sensitivity of 68% and specificity of 62%). For $\dot{V}{\text{O}}_{2\text{peak}}$ estimated using the FitnessGram^®^ equation [15], the optimal cut-point for detecting obesity by combining BMI and WC was 39 mL•kg^-1^•min^-1^ (sensitivity of 73% and specificity of 71%). Table 3, Fig 1 and S4, S5 and S6 Figs show information on AUCs, the optimal cut-points for $\dot{V}{\text{O}}_{2\text{peak}}$ for detecting obesity, and diagnostic measures for each age.

The number of 20mSRT laps achieved showed an AUCs>0.73 to predict obesity estimated by BMI, WC and the combination of BMI and WC for each age in each sex (Table 4, S7, S8, S9, S10, S11, S12 and S13 Figs). When considering the full sample (8–12 years), the optimal cut-point regarding the number of laps to detect obesity estimated by the combination of BMI and WC was 15 in boys and girls.

**Table 4:** Diagnostic properties of 20-meter shuttle run test (maximum number of laps achieved in the test) to detect obesity in boys and girls.

	AUC (95%CI)	Cut-points (Laps)	Sensitivity (%) (95%CI)	Specificity (%) (95%CI)	PPV (%)	NPV (%)	LR+	LR-
**Boys (age)**								
	**Obesity by BMI z-score**							
8	0.74 (0.70–0.78)	16	73.8 (60.9–84.2)	61.7 (57.1–66.3)	20.8	93.8	1.9	0.4
9	0.79 (0.76–0.82)	15	72.2 (64.8–78.8)	73.4 (70.0–76.6)	38.6	91.9	2.7	0.4
10	0.76 (0.73–0.78)	19	76.8 (70.5–82.3)	60.5 (57.2–63.6)	30.7	92.0	1.9	0.4
11	0.80 (0.77–0.82)	18	77.1 (71.7–82.0)	68.2 (65.4–70.9)	36.5	92.6	2.4	0.3
12	0.80 (0.76–0.84)	23	81.6 (71.0–89.5)	63.6 (58.8–68.2)	28.7	95.1	2.2	0.3
8–12	0.78 (0.76–0.79)	17	72.5 (69.2–75.6)	68.9 (67.4–70.4)	33.4	92.1	2.3	0.4
	**Obesity by waist circumference z-score**							
8	0.77 (0.73–0.81)	16	77.1 (59.9–89.5)	60.0 (55.5–64.5)	12.5	97.3	1.9	0.4
9	0.86 (0.84–0.88)	13	90.7 (77.8–97.3)	75.7 (72.7–78.5)	15.8	99.4	3.7	0.1
10	0.81 (0.79–0.83)	17	85.3 (74.6–92.7)	62.7 (59.7–65.6)	12.7	98.5	2.3	0.2
11	0.81 (0.79–0.83)	15	83.1 (71.7–91.2)	71.9 (69.4–74.3)	12.5	98.9	3.0	0.2
12	0.84 (0.80–0.87)	17	82.6 (61.2–94.9)	74.2 (70.0–78.0)	13.4	98.9	3.2	0.2
8–12	0.82 (0.81–0.83)	17	85.5 (80.3–89.7)	64.2 (62.7–65.6)	11.7	98.8	2.4	0.2
	**Obesity by BMI and waist circumference**							
8	0.78 (0.74–0.82)	15	76.9 (56.3–91.0)	62.2 (57.7–66.6)	9.9	98.0	2.0	0.4
9	0.86 (0.84–0.88)	13	90.7 (77.8–97.3)	75.7 (72.7–78.5)	15.8	99.4	3.7	0.1
10	0.82 (0.80–0.84)	17	87.5 (76.8–94.4)	62.6 (59.7–65.5)	12.3	98.8	2.3	0.2
11	0.84 (0.82–0.86)	15	86.9 (75.8–94.1)	71.9 (69.4–74.3)	12.2	99.2	3.1	0.2
12	0.80 (0.76–0.84)	23	81.6 (71.0–89.5)	63.6 (58.8–68.2)	28.7	95.1	2.2	0.3
8–12	0.83 (0.82–0.84)	15	81.9 (76.0–86.8)	69.9 (68.5–71.3)	12.1	98.7	2.7	0.3
**Girls (age)**								
	**Obesity by BMI z-score**							
8	0.69 (0.64–0.73)	12	62.3 (47.9–75.2)	65.6 (61.0–70.0)	17.6	93.6	1.8	0.6
9	0.75 (0.72–0.78)	15	78.9 (71.0–85.5)	60.8 (57.1–64.3)	26.8	94.1	2.0	0.4
10	0.72 (0.69–0.75)	16	80.0 (72.7–86.1)	54.9 (51.7–58.1)	21.7	94.6	1.8	0.4
11	0.74 (0.72–0.76)	17	78.6 (71.6–84.5)	61.1 (58.4–63.8)	20.8	95.6	2.0	0.4
12	0.77 (0.73–0.80)	17	75.5 (61.1–86.6)	67.0 (62.5–71.4)	20.0	96.2	2.3	0.4
8–12	0.73 (0.72–0.75)	15	72.7 (68.8–76.4)	63.6 (62.1–65.2)	22.2	94.2	2.0	0.4
	**Obesity by waist circumference z-score**							
8	0.79 (0.75–0.82)	11	77.8 (52.4–93.5)	73.5 (69.3–77.4)	9.9	98.9	2.9	0.3
9	0.80 (0.77–0.83)	14	81.1 (64.8–92.0)	63.3 (59.9–66.6)	9.0	98.7	2.2	0.3
10	0.76 (0.74–0.79)	13	73.0 (60.3–83.4)	70.9 (68.1–73.6)	13.1	97.8	2.5	0.4
11	0.74 (0.72–0.76)	16	80.3 (69.1–88.8)	62.3 (59.7–64.9)	9.8	98.4	2.1	0.3
12	0.71 (0.67–0.75)	17	70.8 (48.9–87.3)	64.6 (60.1–68.9)	9.2	97.8	2.0	0.5
8–12	0.75 (0.74–0.76)	15	76.5 (70.3–82.0)	60.9 (59.4–62.4)	9.0	98.1	2.0	0.4
	**Obesity by BMI and waist circumference**							
8	0.78 (0.74–0.82)	11	76.5 (50.1–93.0)	73.3 (69.2–77.2)	9.2	98.9	2.9	0.3
9	0.81 (0.78–0.83)	14	84.8 (68.1–94.8)	63.2 (59.8–66.5)	8.4	99.1	2.3	0.2
10	0.76 (0.73–0.78)	13	73.3 (60.3–83.9)	70.8 (67.9–73.5)	12.6	97.9	2.5	0.4
11	0.79 (0.76–0.81)	15	80.7 (68.1–89.9)	66.0 (63.5–68.5)	8.8	98.8	2.4	0.3
12	0.73 (0.69–0.77)	17	75.0 (50.9–91.2)	64.4 (60.0–68.7)	8.1	98.4	2.1	0.4
8–12	0.77 (0.76–0.78)	15	79.7 (73.2–85.2)	60.8 (59.3–62.3)	8.2	98.5	2.0	0.3

BMI: body mass index; AUC: area under the curve; 95% CI: 95% confidence interval; PPV: positive predictive value; NPV: negative predictive value; LR+: positive likelihood ratio; LR: negative likelihood ratio.

The speed (km/h) at the last complete stage duirng the 20mSRT showed an AUCs>0.70 to predict obesity estimated by BMI, WC and the combination of BMI and WC for each age and each sex (Table 5, S14, S15, S16, S17, S18, S19 and S20 Figs). A 20mSRT running speed of 9.0 km/h was the optimal cut-point for detecting obesity and abdominal obesity in both sexes.

**Table 5:** Diagnostic properties of 20-meter shuttle run test (speed for the last complete stage) to detect obesity in boys and girls.

	AUC (95%CI)	Cut-points (km/h)	Sensitivity (%) (95%CI)	Specificity (%) (95%CI)	PPV (%)	NPV (%)	LR+	LR-
**Boys (age)**								
	**Obesity by BMI z-score**							
8	0.71 (0.67–0.75)	9.0	68.9 (55.7–80.1)	64.2 (59.6–68.7)	20.8	93.8	1.9	0.5
9	0.77 (0.74–0.80)	9.0	72.2 (64.8–78.8)	73.4 (70.0–76.6)	38.6	91.9	2.7	0.4
10	0.73 (0.71–0.76)	9.0	60.2 (53.2–66.8)	72.6 (69.6–75.4)	33.3	88.9	2.2	0.6
11	0.77 (0.75–0.80)	9.0	66.1 (60.1–71.7)	77.8 (75.2–80.1)	41.3	90.6	3.0	0.4
12	0.80 (0.76–0.83)	9.5	81.6 (71.0–89.5)	63.6 (58.8–68.2)	28.7	95.1	2.2	0.3
8–12	0.76 (0.74–0.77)	9.0	65.5 (62.0–68.8)	74.4 (73.0–75.9)	35.5	90.9	2.6	0.5
	**Obesity by waist circumference z-score**							
8	0.74 (0.70–0.77)	9.0	71.4 (53.7–85.3)	62.6 (58.0–67.0)	12.4	96.7	1.9	0.5
9	0.83 (0.80–0.85)	9.0	90.7 (77.8–97.3)	67.6 (64.4–70.7)	12.3	99.3	2.8	0.1
10	0.77 (0.75–0.79)	9.0	73.5 (61.4–83.5)	69.0 (66.2–71.8)	13.1	97.6	2.4	0.4
11	0.80 (0.77–0.82)	9.0	83.1 (71.7–91.2)	71.9 (69.4–74.3)	12.5	98.9	3.0	0.2
12	0.82 (0.78–0.85)	9.0	73.9 (51.6–89.7)	78.2 (74.2–81.8)	14.0	98.4	3.4	0.3
8–12	0.79 (0.78–0.80)	9.0	79.1 (73.3–84.1)	70.0 (68.6–71.3)	12.7	98.4	2.6	0.3
	**Obesity by BMI and waist circumference**							
8	0.75 (0.71–0.79)	9.0	76.9 (56.3–91.0)	62.2 (57.7–66.6)	9.9	98.0	2.0	0.4
9	0.83 (0.80–0.85)	9.0	90.7 (77.8–97.3)	67.6 (64.4–70.7)	12.3	99.3	2.8	0.1
10	0.78 (0.76–0.81)	9.0	75.0 (62.6–85.0)	69.0 (66.1–71.7)	12.6	97.9	2.4	0.4
11	0.82 (0.80–0.84)	9.0	86.9 (75.8–94.1)	71.9 (69.4–74.3)	12.2	99.2	3.1	0.2
12	0.82 (0.78–0.85)	9.0	76.2 (52.8–91.7)	78.0 (74.0–81.7)	13.2	98.7	3.5	0.3
8–12	0.80 (0.79–0.82)	9.0	81.9 (76.0–86.8)	69.9 (68.5–71.3)	12.1	98.7	2.7	0.3
**Girls (age)**								
	**Obesity by BMI z-score**							
8	0.66 (0.61–0.70)	9.0	73.6 (59.7–84.7)	52.5 (47.7–57.2)	15.5	94.4	1.6	0.5
9	0.72 (0.69–0.75)	9.0	78.9 (71.0–85.5)	60.8 (57.1–64.3)	26.8	94.1	2.0	0.4
10	0.68 (0.65–0.71)	9.0	74.0 (66.2–80.8)	59.5 (56.3–62.6)	22.2	93.6	1.8	0.4
11	0.72 (0.70–0.75)	9.0	69.6 (62.1–76.5)	68.6 (66.0–71.2)	22.4	94.6	2.2	0.4
12	0.73 (0.69–0.77)	9.0	61.2 (46.2–74.8)	73.9 (69.6–77.9)	20.4	94.6	2.4	0.5
8–12	0.71 (0.69–0.72)	9.0	72.7 (68.8–76.4)	63.6 (62.1–65.2)	22.2	94.2	2.0	0.4
	**Obesity by waist circumference z-score**							
8	0.78 (0.74–0.82)	9.0	88.9 (65.2–98.3)	51.1 (46.6–55.7)	6.3	99.2	1.8	0.2
9	0.76 (0.73–0.79)	9.0	86.5 (71.2–95.4)	56.5 (53.1–59.9)	8.2	98.9	2.0	0.2
10	0.70 (0.68–0.72)	9.0	81.0 (69.1–89.7)	57.1 (54.1–60.2)	10.2	98.0	1.9	0.3
11	0.72 (0.70–0.75)	9.0	73.2 (61.4–83.1)	66.1 (63.6–68.6)	10.0	98.0	2.2	0.4
12	0.69 (0.64–0.73)	9.5	87.5 (67.6–97.2)	43.5 (38.9–48.1)	7.3	98.6	1.6	0.3
8–12	0.72 (0.70–0.73)	9.0	76.5 (70.3–82.0)	60.9 (59.4–62.4)	9.0	98.1	2.0	0.4
	**Obesity by BMI and waist circumference**							
8	0.78 (0.74–0.82)	9.0	88.2 (63.5–98.2)	51.0 (46.5–55.6)	6.0	99.2	1.8	0.2
9	0.77 (0.74–0.80)	9.0	90.9 (75.6–98.0)	56.5 (53.0–59.9)	7.7	99.4	2.1	0.2
10	0.69 (0.66–0.71)	9.0	80.0 (67.7–89.2)	57.0 (53.9–60.0)	9.6	98.0	1.9	0.4
11	0.76 (0.74–0.78)	9.0	80.7 (68.1–89.9)	66.0 (63.5–68.5)	8.8	98.8	2.4	0.3
12	0.69 (0.65–0.73)	9.5	90.0 (68.3–98.5)	43.3 (38.8–47.9)	6.2	99.0	1.6	0.2
8–12	0.73 (0.72–0.74)	9.0	79.7 (73.2–85.2)	60.8 (59.3–62.3)	8.2	98.5	2.0	0.3

BMI: body mass index; AUC: area under the curve; 95% CI: 95% confidence interval; PPV: positive predictive value; NPV: negative predictive value; LR+: positive likelihood ratio; LR: negative likelihood ratio.

According to cut-points suggested in this study for $\dot{V}{\text{O}}_{2\text{peak}}$ (Table 2 for boys, and Table 3 for girls) estimated by the Léger et al. equation [9], FitnessGram^®^ equation [15], number of laps (Table 4), or speed (Table 5), children with low CRF presented higher odds of having obesity (Table 6).

**Table 6:** Association between low levels of cardiorespiratory fitness according to the cut-points of the present study and obesity in boys and girls.

	Boys		Girls	
	Crude Analysis	Adjusted analysis	Crude Analysis	Adjusted analysis
	OR (95% CI)	OR (95% CI)†	OR (95% CI)	OR (95% CI)†
	**Obesity by BMI z-score**			
**Low cardiorespiratory fitness**				
**Sex-age-specific cut-points**				
$\dot{V}{\text{O}}_{2\text{peak}}$—Léger et al.	5.0 (4.3–5.9)*	4.9 (4.1–5.8)*	3.3 (2.8–4.0)*	3.3 (2.8–4.0)*
$\dot{V}{\text{O}}_{2\text{peak}}$—FitnessGram	5.8 (4.9–6.9)*	5.8 (4.8–7.0)*	5.4 (4.4–6.6)*	5.3 (4.3–6.6)*
Laps	5.4 (4.6–6.4)*	5.4 (4.5–6.4)*	4.8 (4.0–5.9)*	4.7 (3.9–5.8)*
Speed	4.1 (3.2–5.2)*	3.7 (2.9–4.8)*	3.3 (2.3–4.9)*	3.0 (2.0–4.4)*
**Sex-specific cut-points**				
$\dot{V}{\text{O}}_{2\text{peak}}$—Léger et al.	5.7 (4.7–6.8)*	6.4 (5.3–7.8)*	2.3 (2.0–2.8)*	4.1 (3.2–5.2)*
$\dot{V}{\text{O}}_{2\text{peak}}$—FitnessGram	5.8 (4.9–6.9)*	6.1 (5.1–7.3)*	5.4 (4.4–6.6)*	4.9 (4.0–6.1)*
Laps	5.6 (4.7–6.6)*	5.7 (4.8–6.8)*	4.4 (3.7–5.3)*	4.3 (3.6–5.2)*
Speed	4.9 (3.7–6.7)*	4.5 (3.3–6.1)*	3.3 (2.3–4.9)*	3.0 (2.0–4.4)*
	**Obesity by waist circumference z-score**			
**Low cardiorespiratory fitness**				
**Sex-age-specific cut-points**				
Léger et al.	5.0 (3.8–6.5)*	4.8 (3.6–6.4)*	3.3 (2.5–4.3)*	3.1 (2.3–4.1)*
FitnessGram	10.2 (7.4–14.1)*	9.8 (6.9–13.8)*	6.4 (4.7–8.7)*	6.2 (4.5–8.7)*
Laps	9.1 (6.7–12.4)*	8.5 (6.1–11.9)*	5.8 (4.3–7.8)*	5.5 (4.0–7.5)*
Speed	5.7 (3.9–8.3)*	5.0 (3.4–7.5)*	3.6 (2.5–5.3)*	3.3 (2.2–4.9)*
**Sex-specific cut-points**				
$\dot{V}{\text{O}}_{2\text{peak}}$—Léger et al.	5.0 (3.7–6.7)*	6.1 (4.3–8.4)*	3.6 (2.7–4.8)*	5.9 (4.0–8.7)*
$\dot{V}{\text{O}}_{2\text{peak}}$—FitnessGram	9.2 (6.5–13.0)*	9.9 (6.8–14.3)*	6.0 (4.5–8.1)*	7.0 (5.0–9.7)*
Laps	9.4 (6.7–13.2)*	9.0 (6.2–12.9)*	4.8 (3.6–6.4)*	4.9 (3.6–6.7)*
Speed	5.7 (3.9–8.3)*	5.0 (3.4–7.5)*	5.6 (3.6–8.7)*	5.2 (3.3–8.2)*
	**Obesity by BMI and waist circumference**			
**Low cardiorespiratory fitness**				
**Sex-age-specific cut-points**				
$\dot{V}{\text{O}}_{2\text{peak}}$—Léger et al.	5.6 (4.2–7.4)*	5.5 (4.0–7.4)*	3.2 (2.4–4.3)*	3.1 (2.3–4.2)*
$\dot{V}{\text{O}}_{2\text{peak}}$ –FitnessGram	12.0 (8.4–17.2)*	11.6 (7.9–17.0)*	6.6 (4.8–9.2)*	6.2 (4.5–8.7)*
Laps	10.1 (7.2–14.2)*	9.6 (6.7–13.8)*	6.3 (4.5–8.6)*	5.9 (4.2–8.1)*
Speed	5.9 (4.0–8.6)*	5.0 (3.4–7.5)*	3.2 (2.1–4.8)*	2.9 (1.9–4.5)*
**Sex-specific cut-points**				
$\dot{V}{\text{O}}_{2\text{peak}}$—Léger et al.	6.0 (4.3–8.4)*	7.5 (5.2–10.8)*	3.4 (2.5–4.6)*	6.0 (4.0–9.1)*
$\dot{V}{\text{O}}_{2\text{peak}}$—FitnessGram	11.8 (7.9–17.7)*	13.1 (8.5–20.3)*	6.4 (4.6–8.8)*	7.6 (5.3–10.9)*
Laps	10.1 (7.3–13.9)*	9.7 (6.9–13.8)*	6.0 (4.3–8.3)*	6.1 (4.3–8.6)*
Speed	5.9 (4.0–8.6)*	5.1 (3.4–7.5)*	4.6 (2.8–7.5)*	4.0 (2.4–6.7)*

OR: odds ratio; 95% CI: 95% confidence interval;

^†^Adjusted analyses for age, site, screen time, and physical activity;

*Logistic regression (p < 0.01).

## Discussion

A number of studies have investigated the relationship between CRF and obesity [34–36] and have identified that low CRF is an independent risk factor for the development of obesity in children and adolescents. These studies have shown that, regardless of the test used to estimate CRF (field or laboratory tests) and body fat (anthropometry, densitometric and/or imaging techniques), these variables were inversely related [34–36]. The present study corroborates these findings and demonstrated that CRF estimated by the 20mSRT was inversely related to obesity estimated by BMI, WC or by the combination of both.

A number of equations have been proposed to estimate $\dot{V}{\text{O}}_{2\text{peak}}$ from the 20mSRT [14–18,37]. Many equations take into account some body fat indicator such as BMI to estimate peak oxygen uptake ($\dot{V}{\text{O}}_{2\text{peak}}$). This strategy was discussed by Cureton and Mahar [19], who recommended that prediction equations should not take into account body fat or physical growth indicators (e.g., height, weight) because of the risk of collinearity. The present study decided to compare the discriminatory ability of $\dot{V}{\text{O}}_{2\text{peak}}$ for obesity estimated by the initial equation of the 20mSRT, with the current equation of the FitnessGram^®^ [15]. The results demonstrated that $\dot{V}{\text{O}}_{2\text{peak}}$ estimated by both equations was adequate to predict obesity in Canadian children. The $\dot{V}{\text{O}}_{2\text{peak}}$ estimated using the FitnessGram^®^ equation [15] presented somewhat better discriminatory power for obesity in most age groups than that estimated using the Léger et al. [9] equation. This difference can be justified by the fact that the sample obtained from the current FitnessGram^®^ equation [15,19] comprises children and adolescents from the last decade and that the sample from the equation of Léger et al. [9] corresponds to earlier decades. It is possible that declining trends in CRF and increasing levels of obesity has had an impact on the accuracy of different equations to predict $\dot{V}{\text{O}}_{2\text{peak}}$ among present day children and youth, which may have had an impact on our ROC curve analysis [3,38]. As the sample of the present study has temporality closer to the sample from the FitnessGram^®^ equation [15], this fact may explain the results observed.

A systematic review and meta-analysis that included 9,280 children and adolescents (49% girls) aged 8–19 years from 14 countries reported CRF cut-point values of 35 and 42 mL•kg^-1^•min^-1^ for girls and boys, respectively, with children and youth falling below these values being at increased risk of cardiovascular disease risk [6]. In the present study, it was observed that CRF values of 39 and 41 mL•kg^-1^•min^-1^ for girls and boys, respectively, could help identify children at risk of obesity, estimated by the combination of BMI and WC. These differences in cut-off values are likely due to the present study only using anthropometric measures of body composition, whereas the Ruiz cut-points used other cardiovascular risk markers [6].

The cut-points proposed by the FitnessGram^®^ for the 20mSRT were developed with statistical procedures similar to the present study, in which the use of the ROC curve served to establish the reference values [39]. In FitnessGram^®^, metabolic syndrome was used as the outcome and the cut-points of $\dot{V}{\text{O}}_{2\text{peak}}$ to classify children within health zones and were determined from 10 years of age (girls—10 years: 40.2 mL•kg^-1^•min^-1^; 11 years: 40.2 mL•kg^-1^•min^-1^, 12 years: 40.1 mL•kg^-1^•min^-1^, boys—10 years: 40.2 mL•kg^-1^•min^-1^, 11 years: 40.2 mL•kg^-1^•min^-1^; 12 years: 40.3 mL•kg^-1^•min^-1^) [39]. The results of the present study demonstrated that, regardless of the equation used to estimate $\dot{V}{\text{O}}_{2\text{peak}}$ (Léger et al. [9] or FitnessGram^®^ [15]), the cut-points to discriminate obesity approached FitnessGram^®^ cut-points [39] and that small differences may be the result of different variables considered as outcome.

Another systematic review that analyzed data from 50 countries proposed normative values for the 20mSRT in children aged 9–17 years [3]. In the criterion-referenced analysis of the present study, it was possible to identify that the 20mSRT running speed at the last complete stage of 9.0 km/h was the cut-point to discriminate obesity in boys and girls aged 8–12 years. This value corresponded to the 10^th^ percentile using the international norms, and reinforces that the last complete stage in the 20mSRT is an indicator capable of predicting increased health risk [3]. In the present study, the cut-points for the number of laps performed in the test varied according to age and sex; however, values above 15 laps in both sexes were considered capable of predicting obesity in all age groups and in both sexes. For boys, this result is close to the normative value of the 10^th^ percentile published in a recent systematic review [3]. On the other hand, for girls, the value found in the present study approaches the 20^th^ percentile reported in the review by Tomkinson et al. [3].

The cut-points found in the present study were used to classify the sample by indicators of obesity in relation to the level of CRF. When using age- and sex-specific cut-points or only sex-specific cut-points, it was found that regardless of the 20mSRT indicator ($\dot{V}{\text{O}}_{2\text{peak}}$, laps, speed), individuals with values below the recommendations were more likely to be obese either by BMI, WC or both, regardless of factors such as age, city of residence, screen time and level of physical activity. This result corroborates other studies that reported CRF as an independent risk factor for obesity [34–36]. The ORs from classifications that considered age- and sex-specific cut-points for CRF were lower than the classifications that considered only sex-specific cut-points. These differences were likely a result of the fact that at each age the cut-points were mostly lower than those found for the full sample. The FitnessGram^®^ battery proposes cut-points specific for each age and sex [39]; the systematic review developed in 50 countries proposed normative values for the 20mSRT specific for each age and sex [11]; and the systematic review that analyzed the 20mSRT with health indicators proposed cut-points specific for each sex [13], without specifying age.

This study has several limitations. First, the sample is non-probabilistic. However, children across many sites in Canada participated in the survey, and a deliberate attempt was made to sample children across socioeconomic and rural/urban strata, and the sample size was very large. Second, indicators analyzed (BMI and WC) are considered less accurate for estimating body fat than skinfolds, densitometric, or imaging techniques to identify obesity [27]. However, such indicators are recommended when it comes to large samples due to low operating cost and easy application. Moreover, the combination of the two indicators used in the present study can be considered an adequate strategy because it classified young people as general and central obesity simultaneously [40]. Third, the cross-sectional design prevents establishing causal relationships between performance on the 20mSRT and obesity, and the possibility of reverse causation is also present. However, evidence from the literature suggests that previous CRF cut-points were also developed using cross-sectional samples [13].

It could be concluded that all indicators of the 20mSRT ($\dot{V}{\text{O}}_{2\text{peak}}$, number of laps, speed) were accurate in identifying obesity in Canadian children aged 8–12 years. $\dot{V}{\text{O}}_{2\text{peak}}$ estimated by the Léger et al. equations [9] and the FitnessGram^®^ equation [15] presented adequate predictive abilities for obesity. However, the FitnessGram^®^ equation [15] presented somewhat higher discriminatory power for obesity than the Léger et al. equation [9]. Regardless of age, place of residence, screen time and level of physical activity, children with low levels of CRF demonstrated greater chances of obesity.

## Supporting information

S1 FigReceiver operating characteristic curve of $\dot{V}{\text{O}}_{2\text{peak}}$ estimated by the Léger et al. [6] and FitnessGram [12] equations to detect obesity by body mass index (BMI) in boys according to age.(TIF)

S2 FigReceiver operating characteristic curve of $\dot{V}{\text{O}}_{2\text{peak}}$ estimated by the Léger et al. [6] and FitnessGram [12] equations to detect obesity by waist circumference (WC) in boys according to age.(TIF)

S3 FigReceiver operating characteristic curve of $\dot{V}{\text{O}}_{2\text{peak}}$ estimated by the Léger et al. [6] and FitnessGram [12] equations to detect obesity by combination of the body mass index (BMI) and waist circumference (WC) in boys according to age.(TIF)

S4 FigReceiver operating characteristic curve of $\dot{V}{\text{O}}_{2\text{peak}}$ estimated by the Léger et al. [6] and FitnessGram [12] equations to detect obesity by body mass index (BMI) in girls according to age.(TIF)

S5 FigReceiver operating characteristic curve of $\dot{V}{\text{O}}_{2\text{peak}}$ estimated by the Léger et al. [6] and FitnessGram [12] equations to detect obesity by waist circumference (WC) in girls according to age.(TIF)

S6 FigReceiver operating characteristic curve of $\dot{V}{\text{O}}_{2\text{peak}}$ estimated by the Léger et al. [6] and FitnessGram [12] equations to detect obesity by combination of the body mass index (BMI) and waist circumference (WC) in girls according to age.(TIF)

S7 Fig Receiver operating characteristic curve of maximum number of laps achieved in the 20-meter shuttle run test to detect obesity in boys and girls according to body mass index (BMI), waist circumference (WC), and the combination between BMI and WC. (TIF)

S8 Fig Receiver operating characteristic curve of maximum number of laps achieved in the 20-meter shuttle run test to detect obesity by body mass index (BMI) in boys according to age. (TIF)

S9 Fig Receiver operating characteristic curve of maximum number of laps achieved in the 20-meter shuttle run test to detect obesity by body mass index (BMI) in girls according to age. (TIF)

S10 Fig Receiver operating characteristic curve of maximum number of laps achieved in the 20-meter shuttle run test to detect obesity by waist circumference (WC) in boys according to age. (TIF)

S11 Fig Receiver operating characteristic curve of maximum number of laps achieved in the 20-meter shuttle run test to detect obesity by waist circumference (WC) in girls according to age. (TIF)

S12 Fig Receiver operating characteristic curve of maximum number of laps achieved in the 20-meter shuttle run test to detect obesity by combination of body mass index (BM) and waist circumference (WC) in boys according to age. (TIF)

S13 Fig Receiver operating characteristic curve of maximum number of laps achieved in the 20-meter shuttle run test to detect obesity by combination of body mass index (BM) and waist circumference (WC) in girls according to age. (TIF)

S14 Fig Receiver operating characteristic curve of speed for the last complete stage in the 20-meter shuttle run test to detect obesity in boys and girls according to body mass index (BMI), waist circumference (WC), and the combination between BMI and WC. (TIF)

S15 Fig Receiver operating characteristic curve of speed for the last complete stage in the 20-meter shuttle run test to detect obesity by body mass index (BMI) in boys according to age. (TIF)

S16 Fig Receiver operating characteristic curve of speed for the last complete stage in the 20-meter shuttle run test to detect obesity by body mass index (BMI) in girls according to age. (TIF)

S17 Fig Receiver operating characteristic curve of speed for the last complete stage in the 20-meter shuttle run test to detect obesity by waist circumference (WC) in boys according to age. (TIF)

S18 Fig Receiver operating characteristic curve of speed for the last complete stage in the 20-meter shuttle run test to detect obesity by waist circumference (WC) in girls according to age. (TIF)

S19 Fig Receiver operating characteristic curve of speed for the last complete stage in the 20-meter shuttle run test to detect obesity by combination of body mass index (BM) and waist circumference (WC) in boys according to age. (TIF)

S20 Fig Receiver operating characteristic curve of speed for the last complete stage in the 20-meter shuttle run test to detect obesity by combination of body mass index (BM) and waist circumference (WC) in girls according to ages. (TIF)

S1 Table Pearson correlation coefficient (r) of association between 20-shuttle run test indicators and body mass index and waist circumference in Canadian children. BMI: body mass index; WC: waist circumference; **p < 0.01 (Pearson correlation coefficient—r).
(DOCX)
