# Supplementary figures and images for: Cardiorespiratory fitness in children: Evidence for criterion-referenced cut-points
Source: PLoS One. 2018 Aug 1;13(8):e0201048. doi: 10.1371/journal.pone.0201048 (PMC6070257; doi:10.1371/journal.pone.0201048)

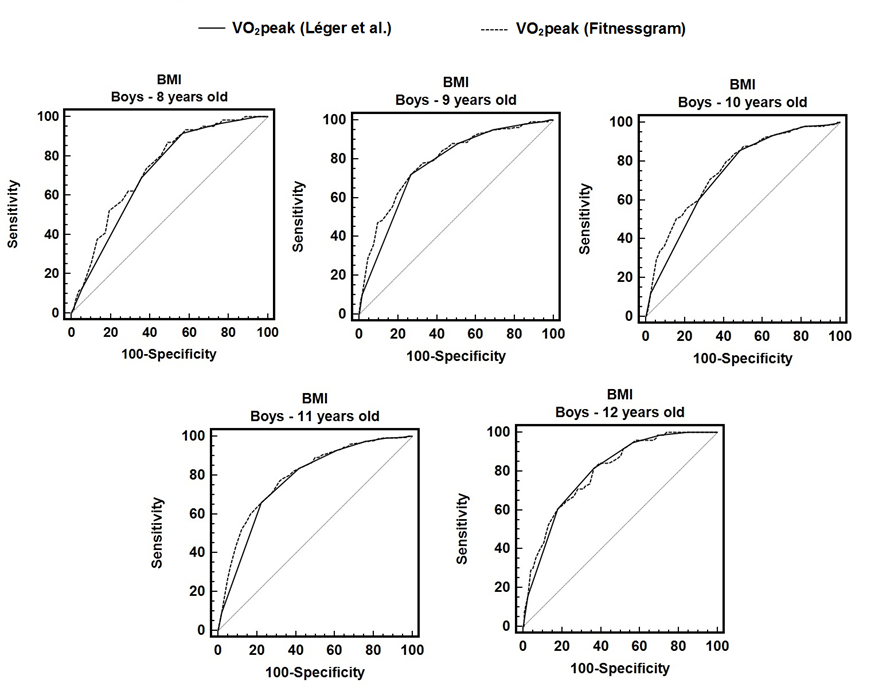

Supplement: S1 Fig — (TIF) [file pone.0201048.s001.tif]

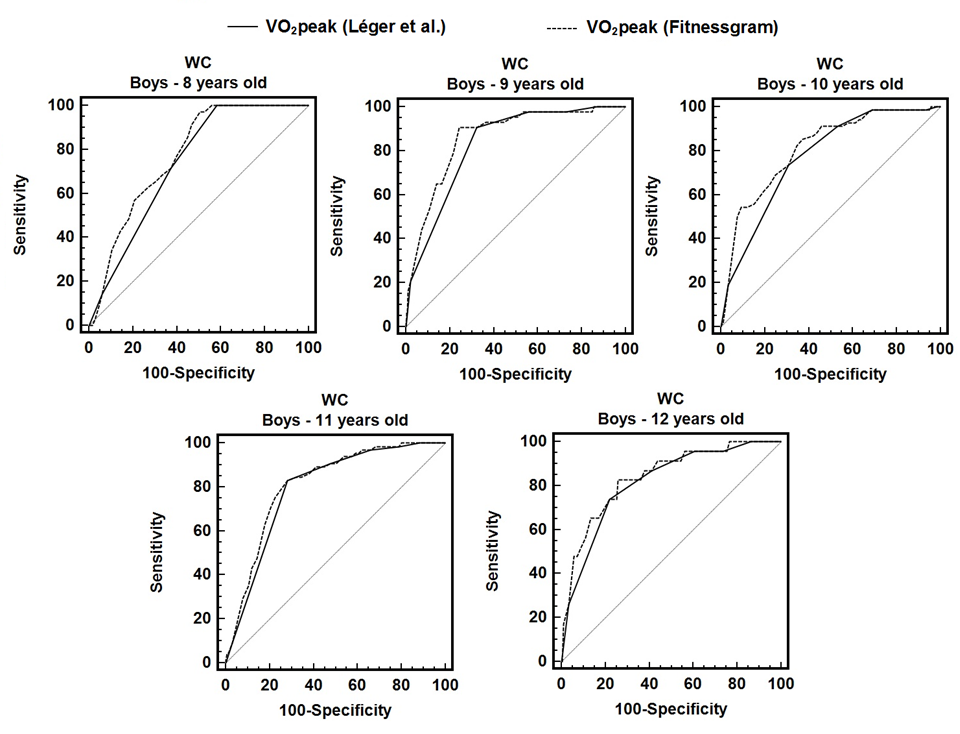

Supplement: S2 Fig — (TIF) [file pone.0201048.s002.tif]

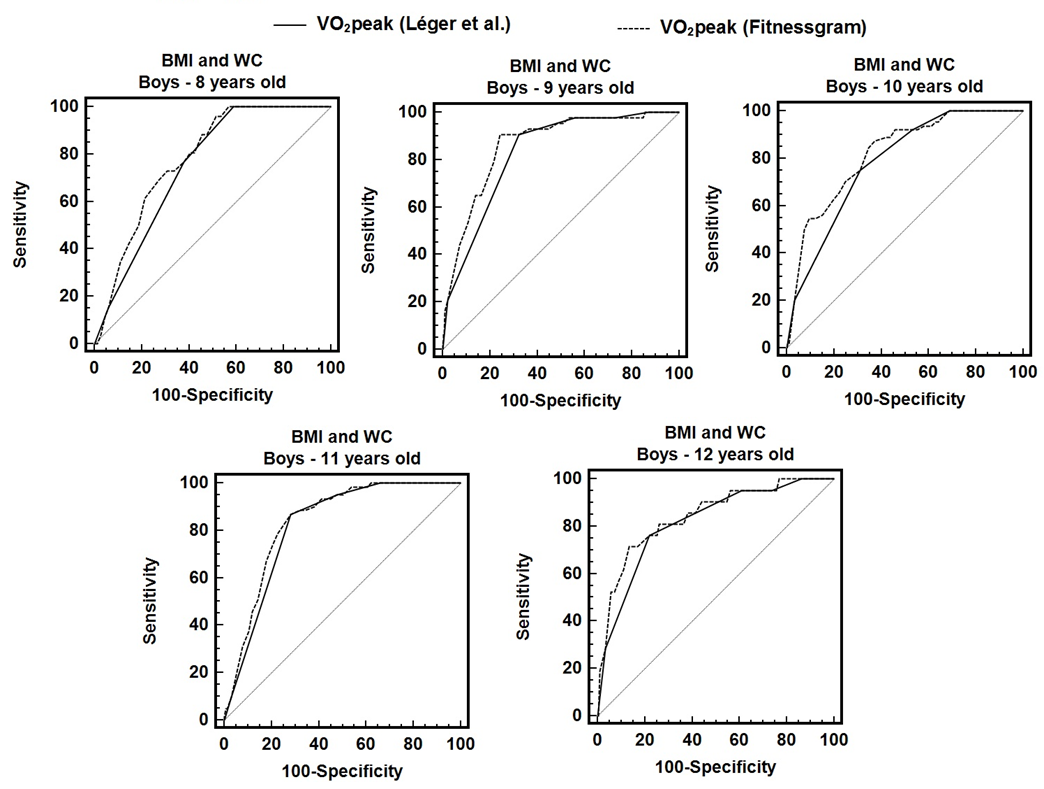

Supplement: S3 Fig — (TIF) [file pone.0201048.s003.tif]

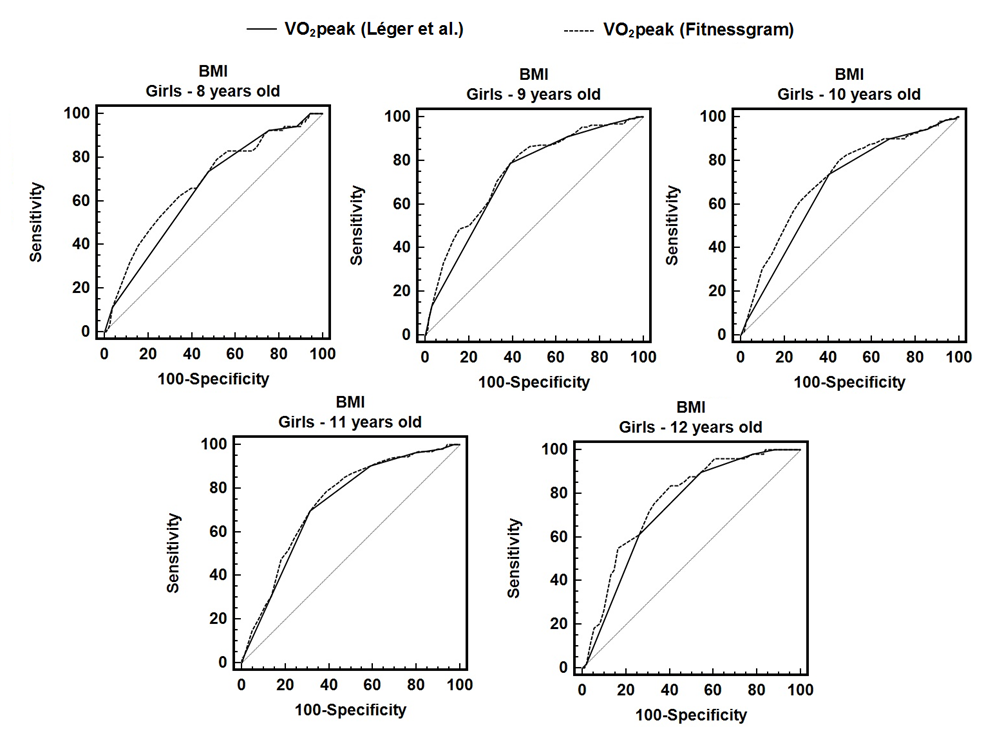

Supplement: S4 Fig — (TIF) [file pone.0201048.s004.tif]

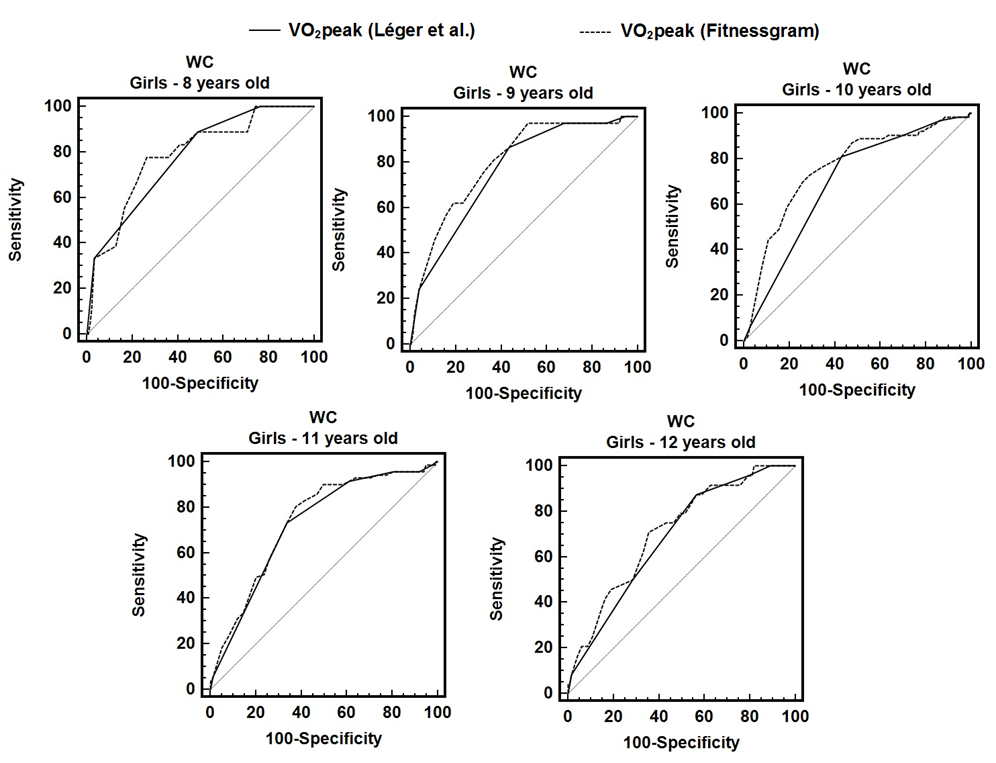

Supplement: S5 Fig — (TIF) [file pone.0201048.s005.tif]

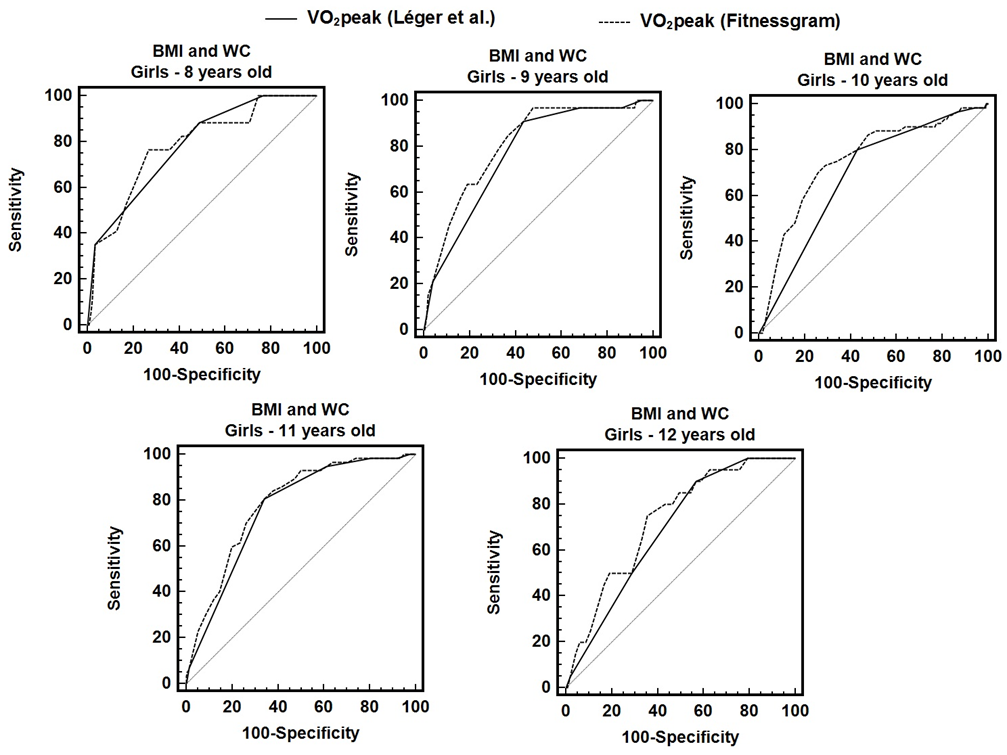

Supplement: S6 Fig — (TIF) [file pone.0201048.s006.tif]

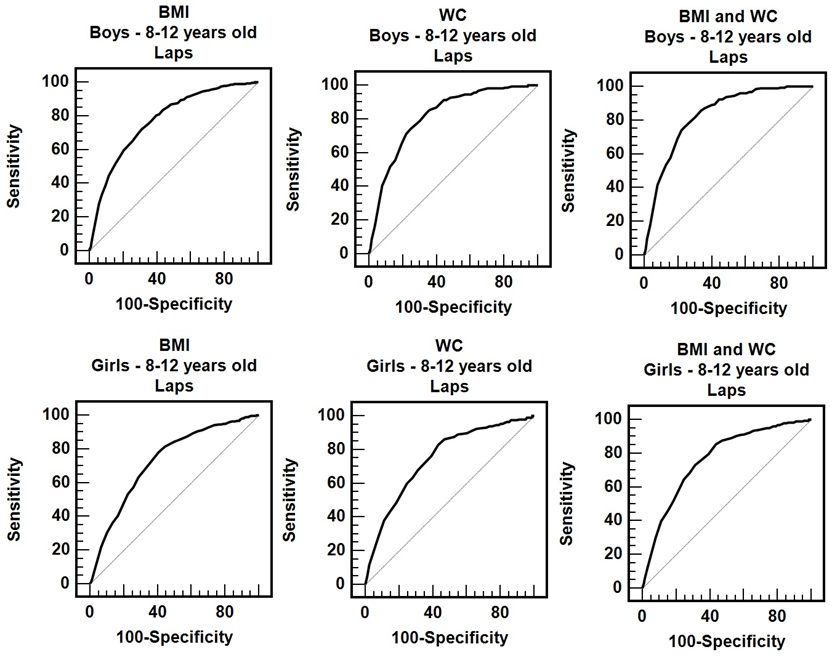

Supplement: S7 Fig — (TIF) [file pone.0201048.s007.tif]

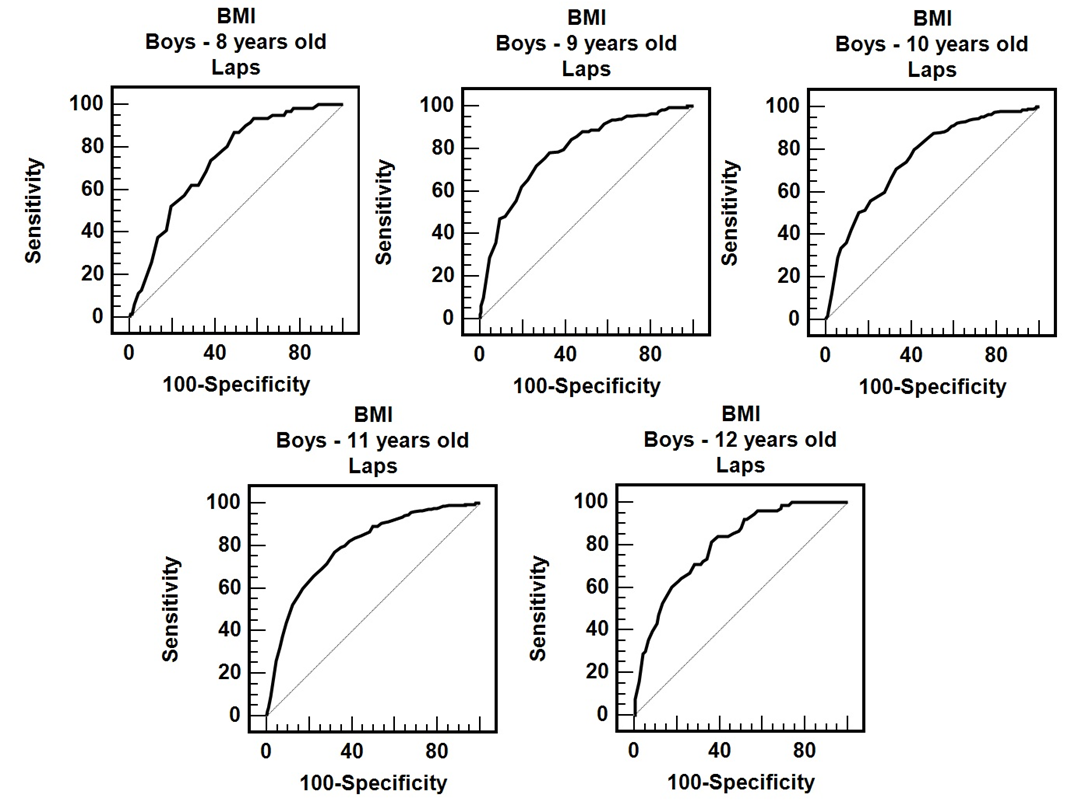

Supplement: S8 Fig — (TIF) [file pone.0201048.s008.tif]

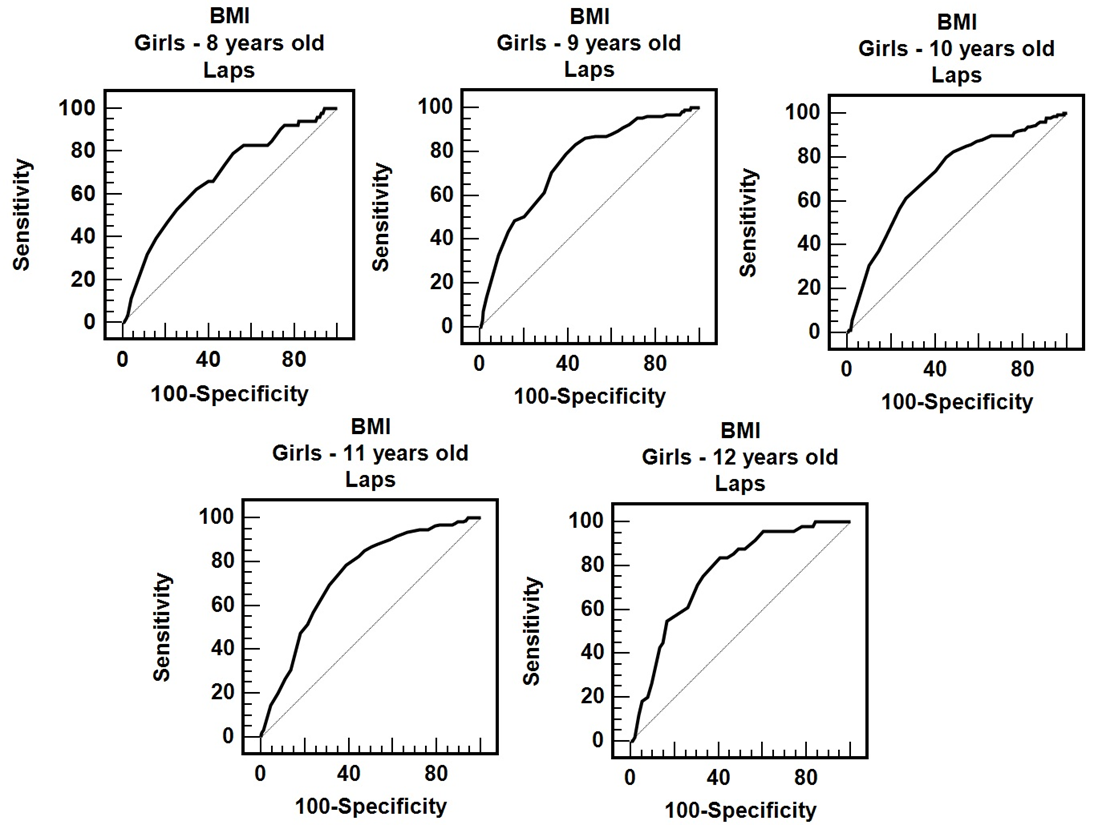

Supplement: S9 Fig — (TIF) [file pone.0201048.s009.tif]

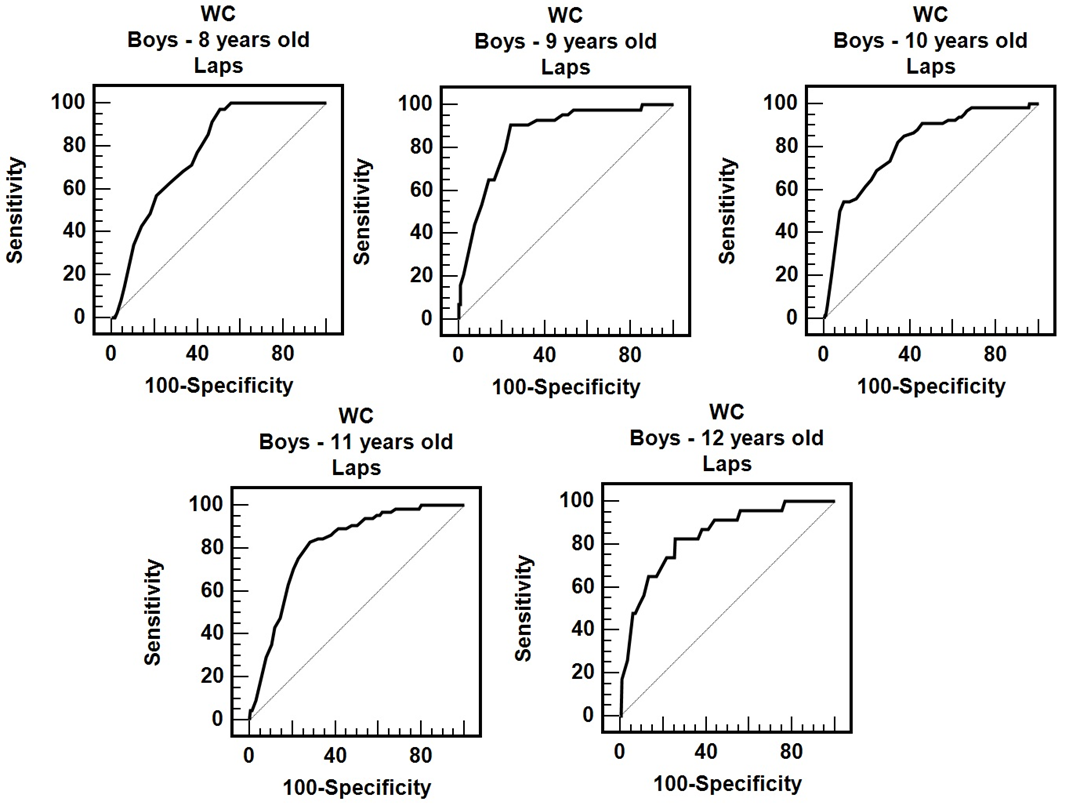

Supplement: S10 Fig — (TIF) [file pone.0201048.s010.tif]

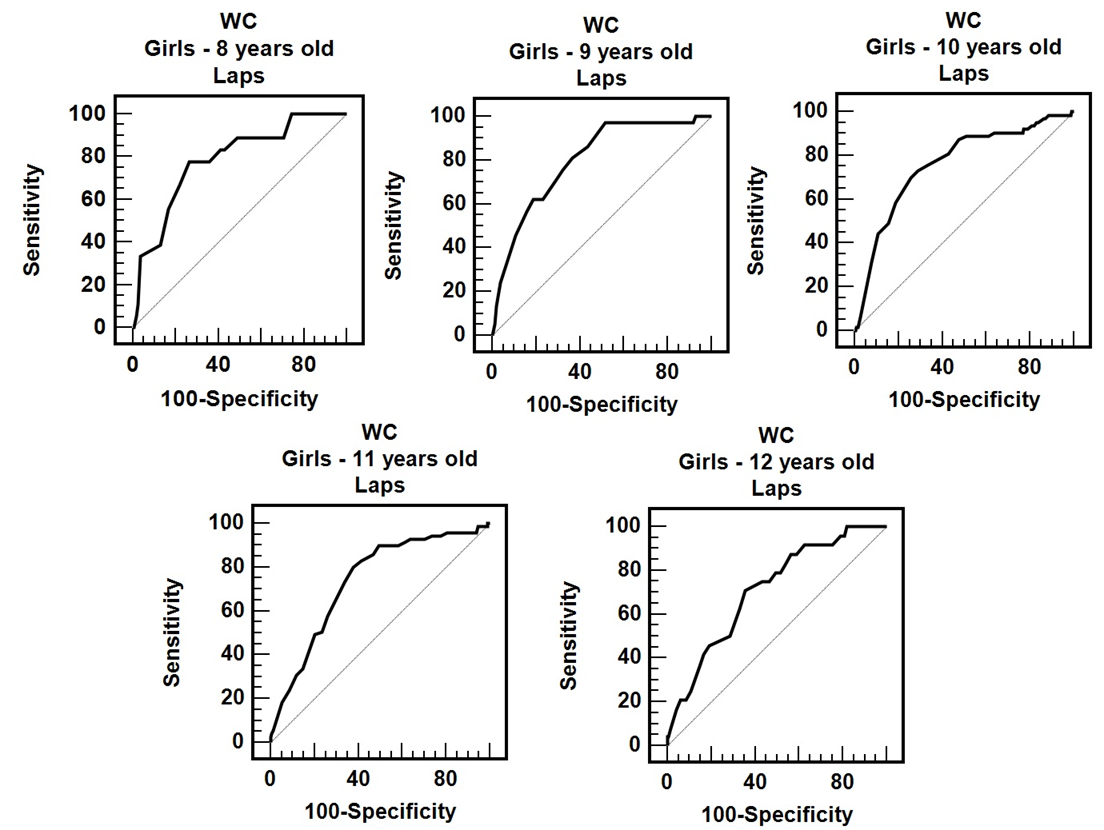

Supplement: S11 Fig — (TIF) [file pone.0201048.s011.tif]

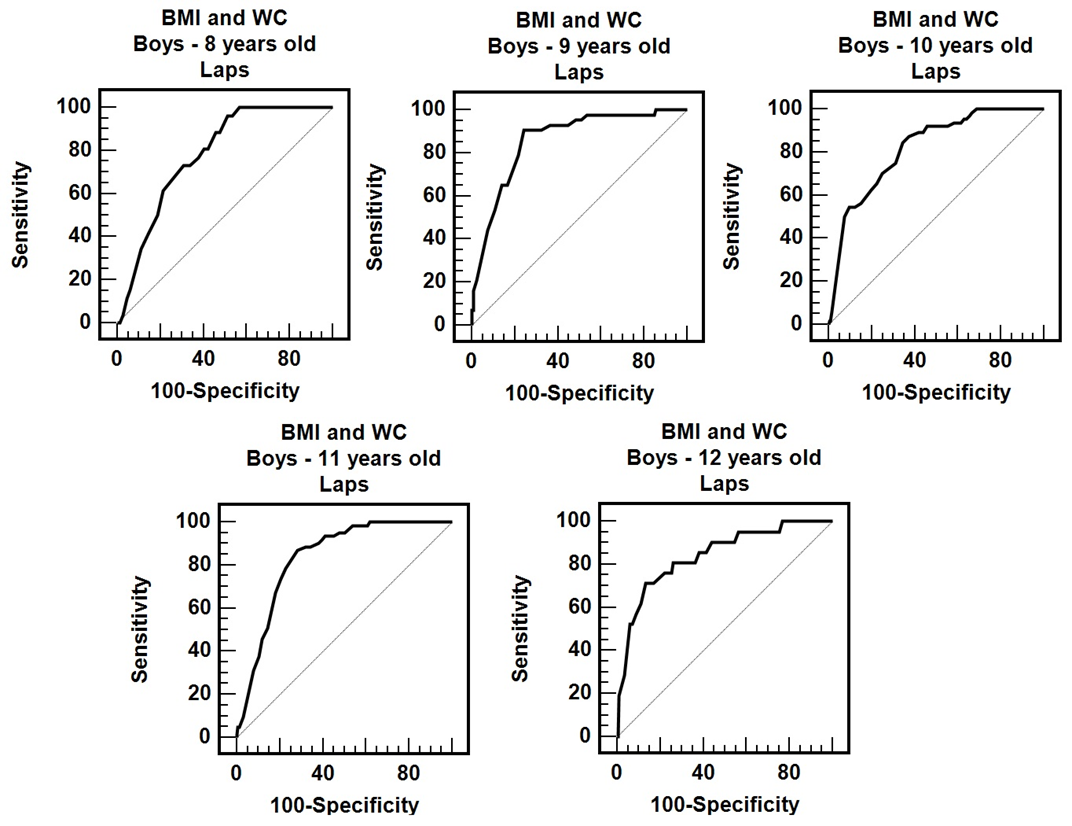

Supplement: S12 Fig — (TIF) [file pone.0201048.s012.tif]

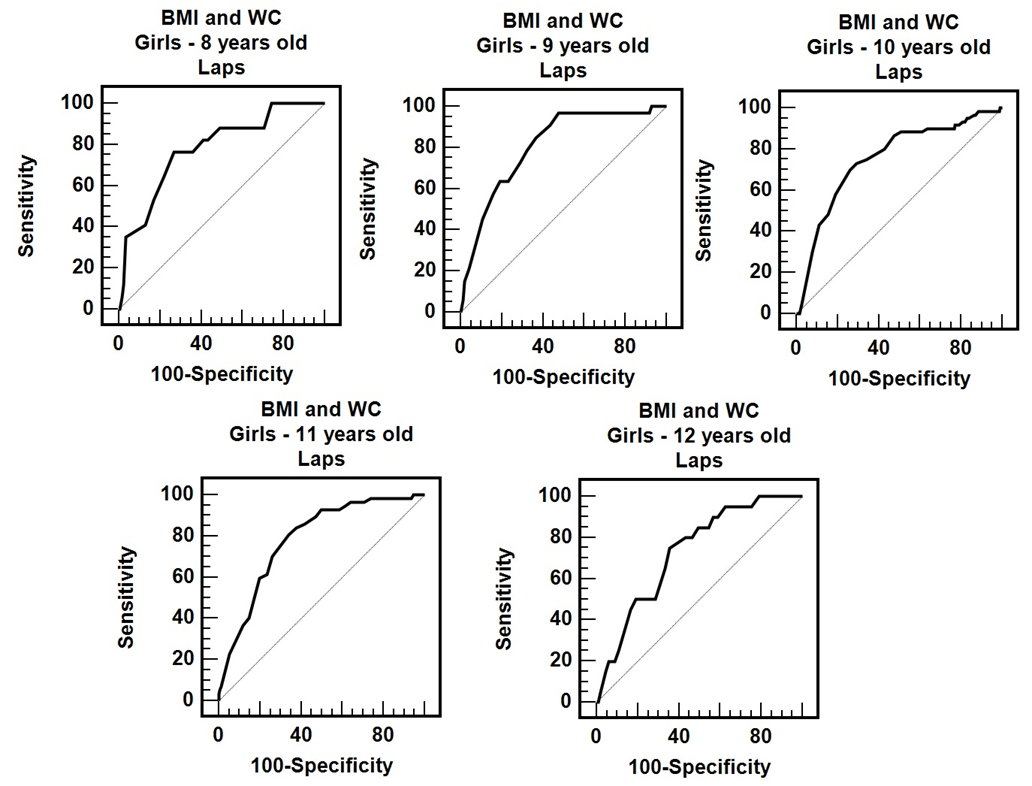

Supplement: S13 Fig — (TIF) [file pone.0201048.s013.tif]

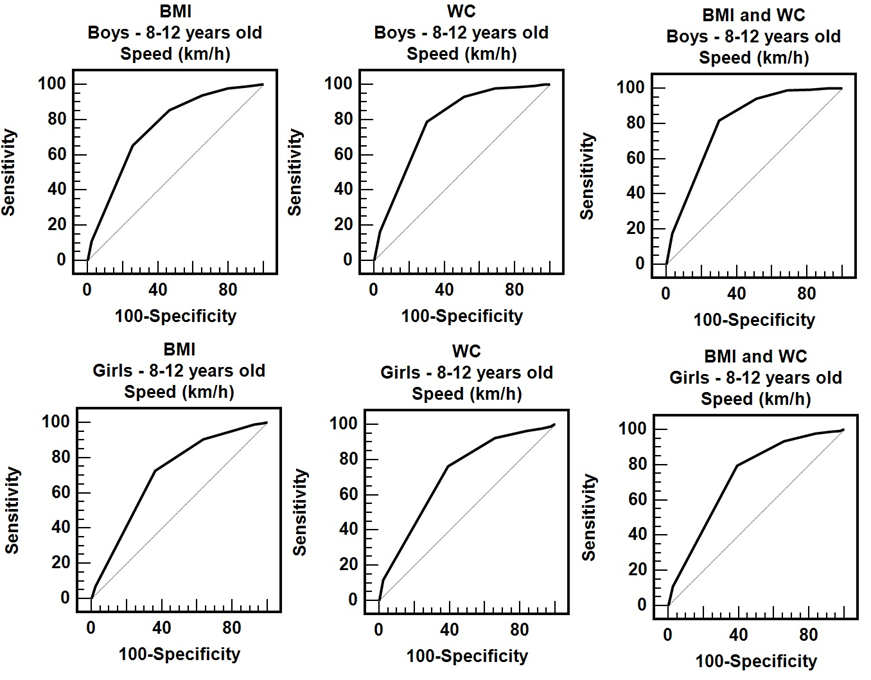

Supplement: S14 Fig — (TIF) [file pone.0201048.s014.tif]

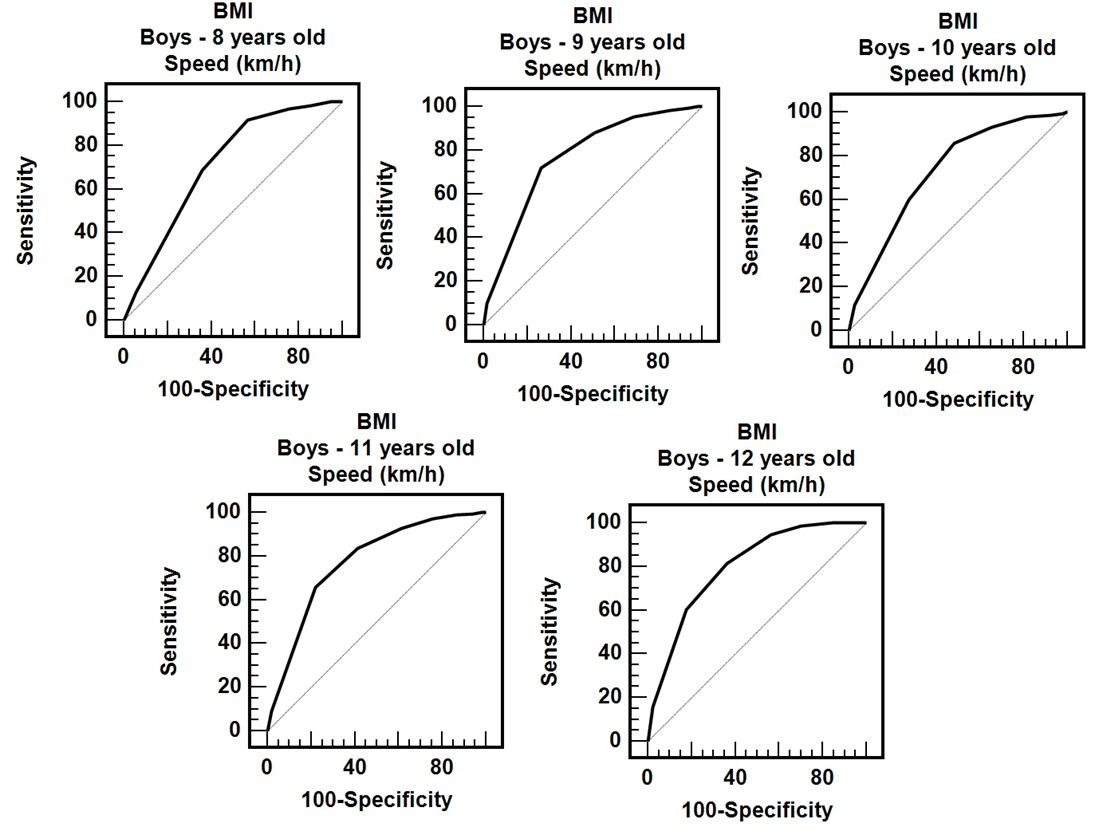

Supplement: S15 Fig — (TIF) [file pone.0201048.s015.tif]

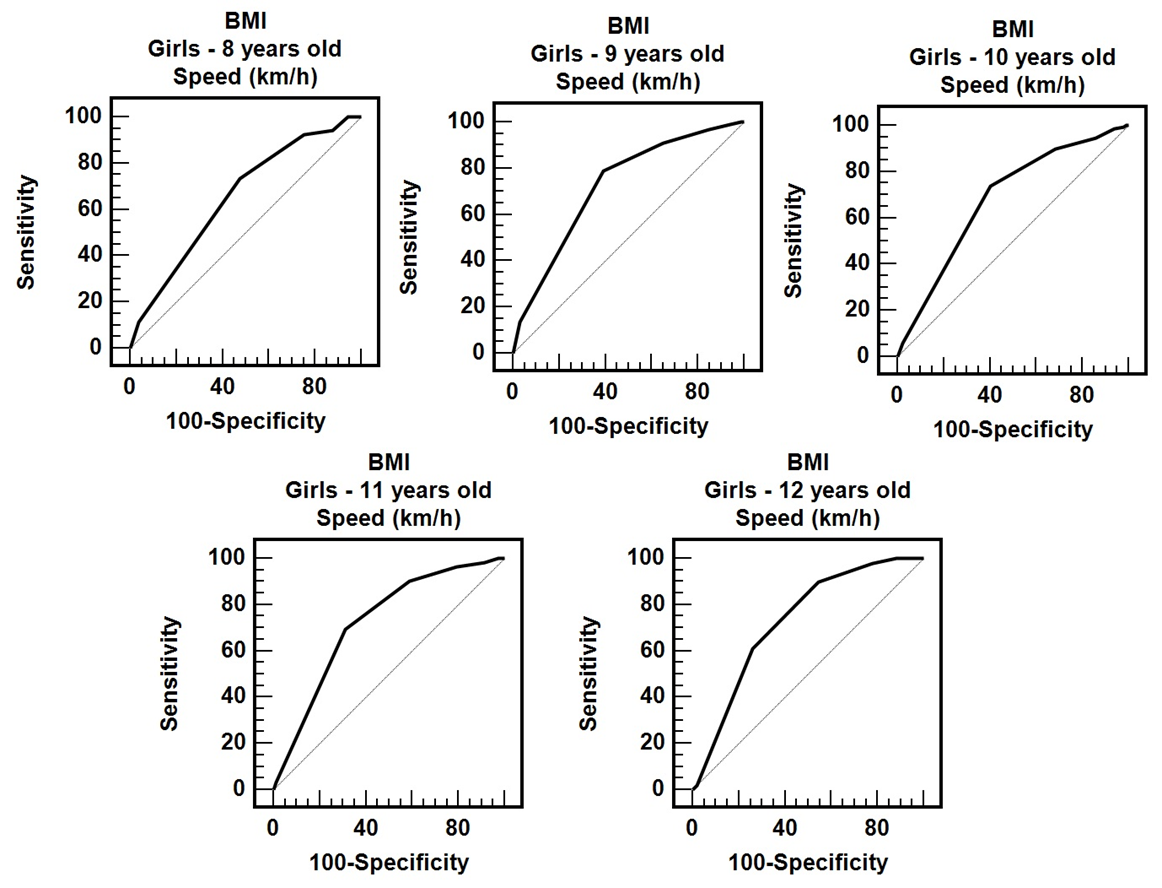

Supplement: S16 Fig — (TIF) [file pone.0201048.s016.tif]

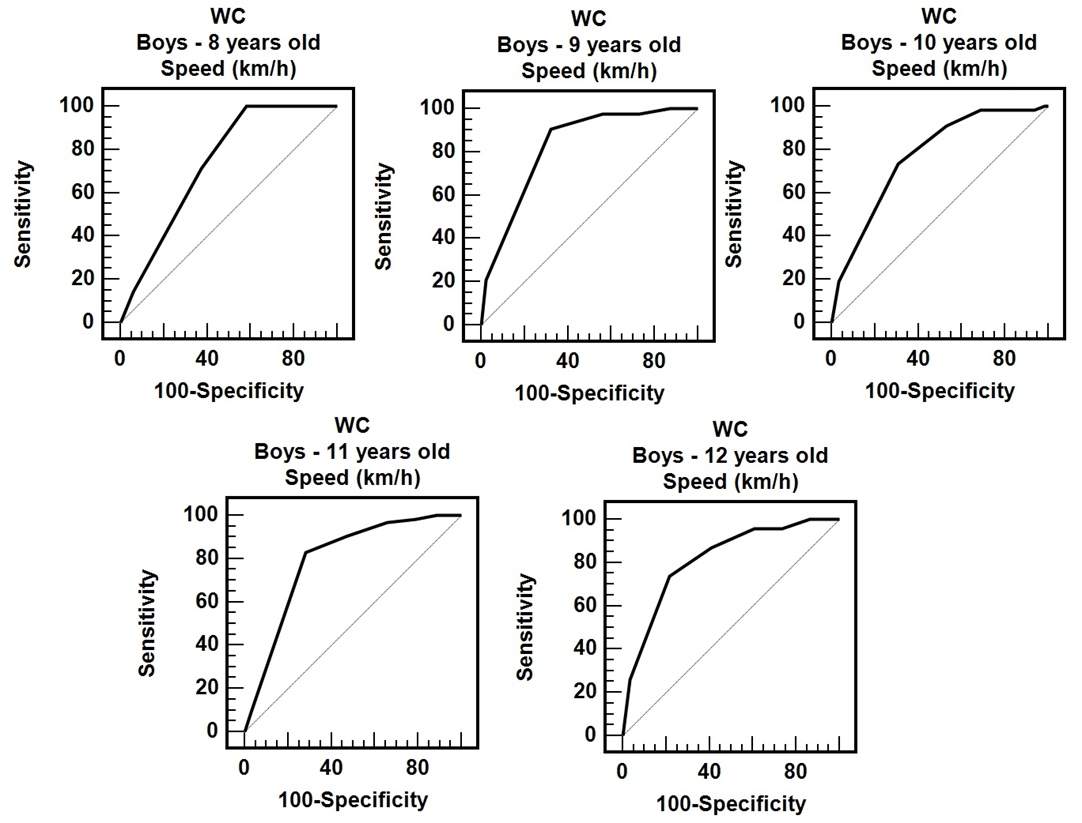

Supplement: S17 Fig — (TIF) [file pone.0201048.s017.tif]

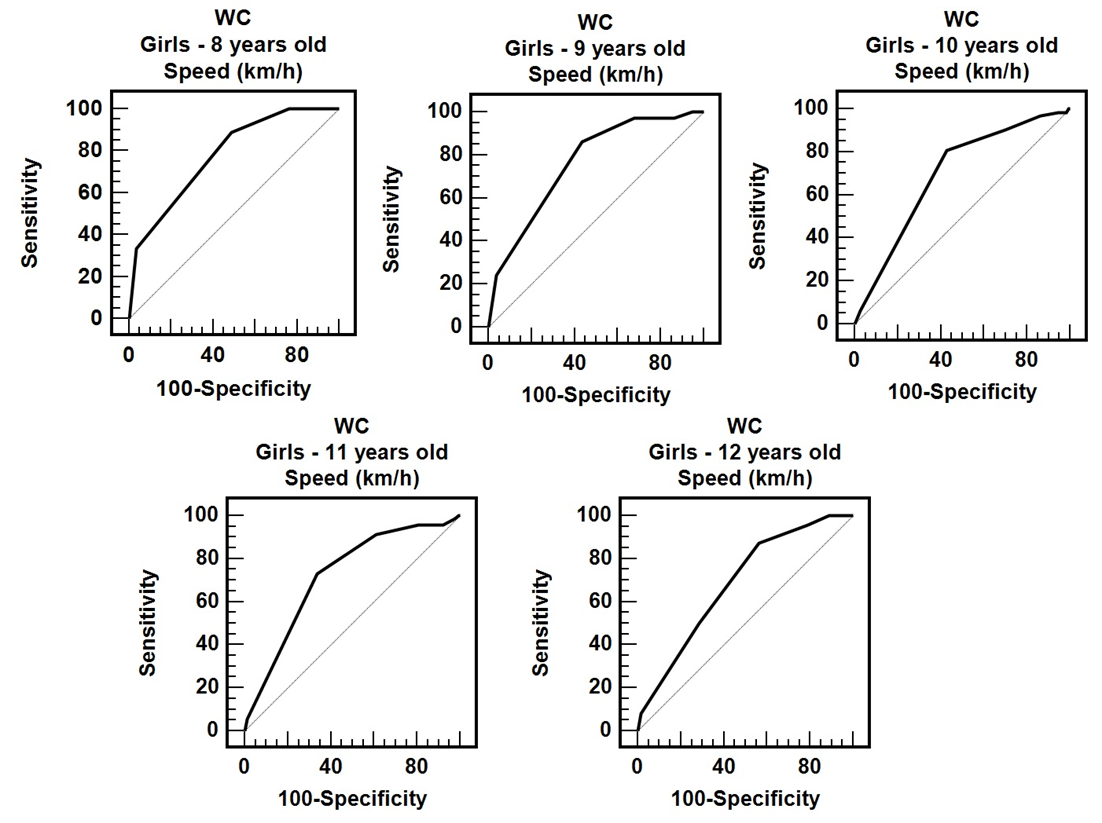

Supplement: S18 Fig — (TIF) [file pone.0201048.s018.tif]

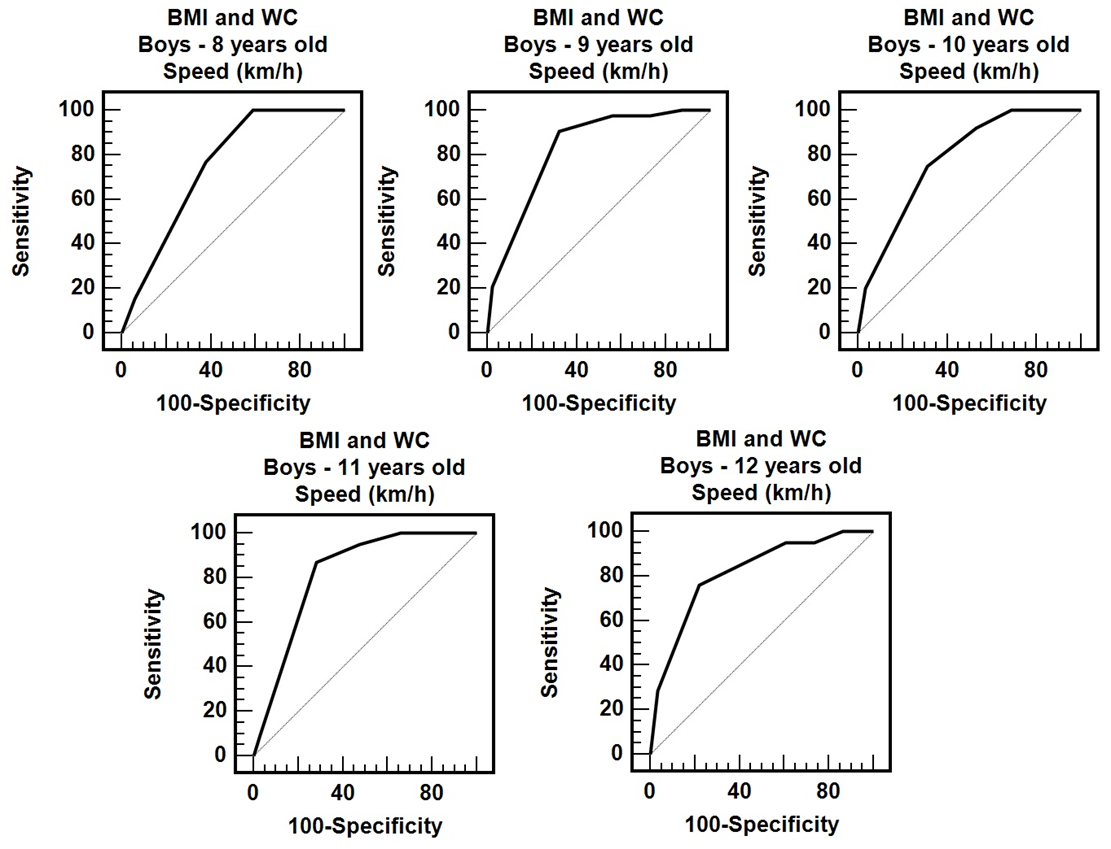

Supplement: S19 Fig — (TIF) [file pone.0201048.s019.tif]

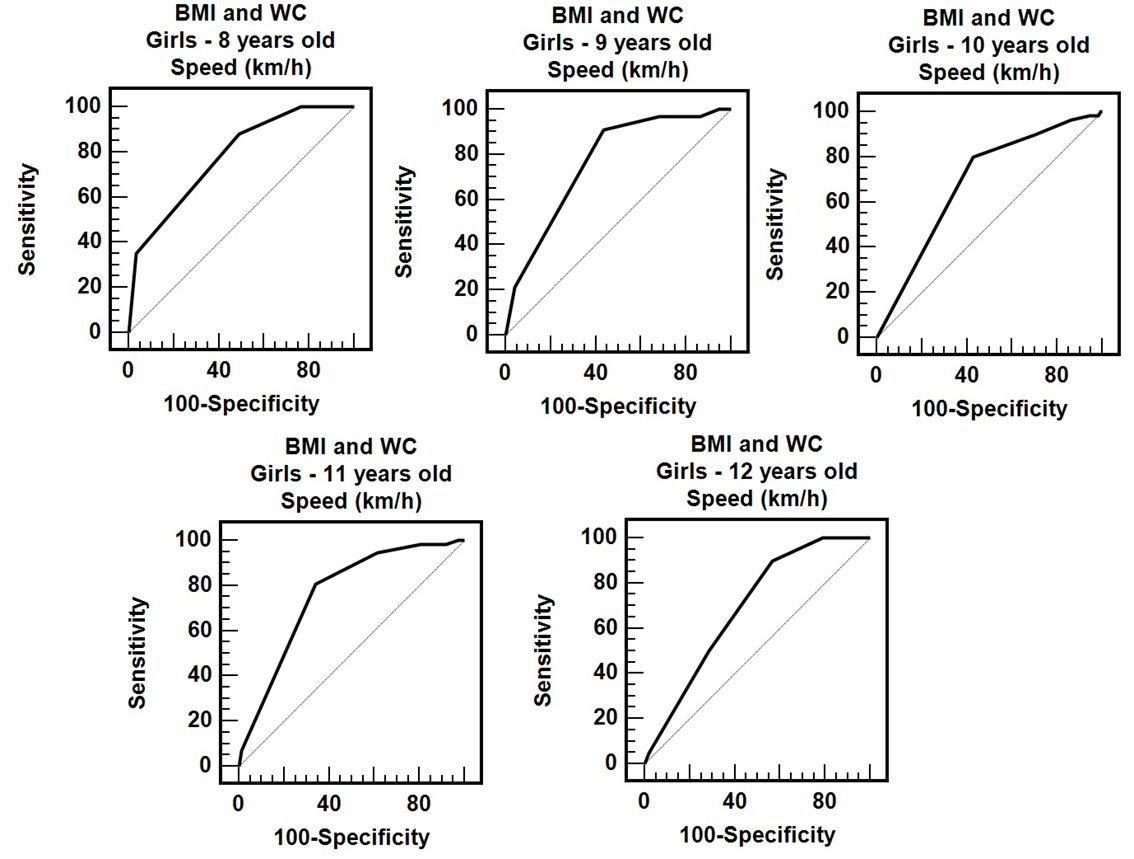

Supplement: S20 Fig — (TIF) [file pone.0201048.s020.tif]
