# Supplementary material for: Cardiorespiratory fitness in children: Evidence for criterion-referenced cut-points
Source: PLoS One. 2018 Aug 1;13(8):e0201048. doi: 10.1371/journal.pone.0201048 (PMC6070257; doi:10.1371/journal.pone.0201048)
Supplement: S1 Table — BMI: body mass index; WC: waist circumference; **p < 0.01 (Pearson correlation coefficient—r). (DOCX) [file pone.0201048.s021.docx]

**Table S1**. Pearson correlation coefficient (r) of association between 20-shuttle run test indicators and body mass index and waist circumference in Canadian children.

|  | $\dot{\boldsymbol{V}}$**O_2peak_**  **(mL•kg^-1^•min^-1^)**  **(Léger et al.)** | $\dot{\boldsymbol{V}}$**O_2peak_**  **(mL•kg^-1^•min^-1^)**  **(FitnessGram)** | **Laps (n)** | **Last complete stage (n)** | **Speed (km/h)**  **(last complete stage)** |
| --- | --- | --- | --- | --- | --- |
|  | **Boys (8 years old)** | | | | |
| **BMI (kg/m²)** | –0.21** | –0.22** | –0.22** | –0.22** | –0.21** |
| **WC (cm)** | –0.23** | –0.23** | –0.23** | –0.23** | –0.23** |
|  | **Boys (9 years old)** | | | | |
| **BMI (kg/m²)** | –0.37** | –0.36** | –0.36** | –0.37** | –0.37** |
| **WC (cm)** | –0.35** | –0.36** | –0.36** | –0.37** | –0.35** |
|  | **Boys (10 years old)** | | | | |
| **BMI (kg/m²)** | –0.35** | –0.36** | –0.36** | –0.36** | –0.35** |
| **WC (cm)** | –0.37** | –0.37** | –0.37** | –0.38** | –0.37** |
|  | **Boys (11 years old)** | | | | |
| **BMI (kg/m²)** | –0.39** | –0.39** | –0.39** | –0.40** | –0.39** |
| **WC (cm)** | –0.39** | –0.39** | –0.39** | –0.39** | –0.39** |
|  | **Boys (12 years old)** | | | | |
| **BMI (kg/m²)** | –0.40** | –0.39** | –0.39** | –0.39** | –0.40** |
| **WC (cm)** | –0.40** | –0.38** | –0.38** | –0.39** | –0.40** |
|  | **Boys (8-12 years old)** | | | | |
| **BMI (kg/m²)** | –0.41** | –0.38** | –0.32** | –0.33** | –0.33** |
| **WC (cm)** | –0.43** | –0.38** | –0.31** | –0.31** | –0.31** |
|  | **Girls (8 years old)** | | | | |
| **BMI (kg/m²)** | –0.21** | –0.20** | –0.20** | –0.20** | –0.21** |
| **WC (cm)** | –0.24** | –0.23** | –0.23** | –0.23** | –0.24** |
|  | **Girls (9 years old)** | | | | |
| **BMI (kg/m²)** | –0.33** | –0.33** | –0.33** | –0.33** | –0.33** |
| **WC (cm)** | –0.34** | –0.34** | –0.34** | –0.33** | –0.34** |
|  | **Girls (10 years old)** | | | | |
| **BMI (kg/m²)** | –0.29** | –0.31** | –0.31** | –0.30** | –0.29** |
| **WC (cm)** | –0.30** | –0.32** | –0.32** | –0.31** | –0.30** |
|  | **Girls (11 years old)** | | | | |
| **BMI (kg/m²)** | –0.31** | –0.32** | –0.32** | –0.32** | –0.31** |
| **WC (cm)** | –0.34** | –0.34** | –0.34** | –0.34** | –0.34** |
|  | **Girls (12 years old)** | | | | |
| **BMI (kg/m²)** | –0.29** | –0.30** | –0.30** | –0.31** | –0.29** |
| **WC (cm)** | –0.28** | –0.28** | –0.28** | –0.29** | –0.28** |
|  | **Girls (8-12 years old)** | | | | |
| **BMI (kg/m²)** | –0.35** | –0.33** | –0.27** | –0.27** | –0.26** |
| **WC (cm)** | –0.39** | –0.35** | –0.27** | –0.26** | –0.26** |

BMI: body mass index; WC: waist circumference; **p < 0.01 (Pearson correlation coefficient – r).
